# The Functional Consequences of Variation in Transcription Factor Binding

**DOI:** 10.1371/journal.pgen.1004226

**Published:** 2014-03-06

**Authors:** Darren A. Cusanovich, Bryan Pavlovic, Jonathan K. Pritchard, Yoav Gilad

**Affiliations:** 1 Department of Human Genetics, University of Chicago, Chicago, Illinois, United States of America; 2 Howard Hughes Medical Institute, University of Chicago, Chicago, Illinois, United States of America; 3 Departments of Genetics and Biology and Howard Hughes Medical Institute, Stanford University, Stanford, California, United States of America; Weizmann Institute of Science, Israel

## Abstract

One goal of human genetics is to understand how the information for precise and dynamic gene expression programs is encoded in the genome. The interactions of transcription factors (TFs) with DNA regulatory elements clearly play an important role in determining gene expression outputs, yet the regulatory logic underlying functional transcription factor binding is poorly understood. Many studies have focused on characterizing the genomic locations of TF binding, yet it is unclear to what extent TF binding at any specific locus has functional consequences with respect to gene expression output. To evaluate the context of functional TF binding we knocked down 59 TFs and chromatin modifiers in one HapMap lymphoblastoid cell line. We then identified genes whose expression was affected by the knockdowns. We intersected the gene expression data with transcription factor binding data (based on ChIP-seq and DNase-seq) within 10 kb of the transcription start sites of expressed genes. This combination of data allowed us to infer functional TF binding. Using this approach, we found that only a small subset of genes bound by a factor were differentially expressed following the knockdown of that factor, suggesting that most interactions between TF and chromatin do not result in measurable changes in gene expression levels of putative target genes. We found that functional TF binding is enriched in regulatory elements that harbor a large number of TF binding sites, at sites with predicted higher binding affinity, and at sites that are enriched in genomic regions annotated as “active enhancers.”

## Author Summary

An important question in genomics is to understand how a class of proteins called “transcription factors” controls the expression level of other genes in the genome in a cell-type-specific manner – a process that is essential to human development. One major approach to this problem is to study where these transcription factors bind in the genome, but this does not tell us about the effect of that binding on gene expression levels and it is generally accepted that much of the binding does not strongly influence gene expression. To address this issue, we artificially reduced the concentration of 59 different transcription factors in the cell and then examined which genes were impacted by the reduced transcription factor level. Our results implicate some attributes that might influence what binding is functional, but they also suggest that a simple model of functional vs. non-functional binding may not suffice.

## Introduction

Understanding the regulatory logic of the genome is critical to understanding human biology. Ultimately, we aim to be able to predict the expression pattern of a gene based on its regulatory sequence alone. However, the regulatory code of the human genome is much more complicated than the triplet code of protein coding sequences, and is highly context-specific, depending on cell-type and other factors [1]. In addition, regulatory regions are not necessarily organized into discrete, easily identifiable regions of the genome and may exert their influence on genes over large genomic distances [2]. Consequently, the rules governing the sequence specificity as well as the functional output of even the most common regulatory interactions, such as interactions between transcription factors and the genome, are not yet fully understood.

To date, genomic studies addressing questions of the regulatory logic of the human genome have largely taken one of two approaches. On the one hand are studies aimed at collecting transcription factor binding maps using techniques such as ChIP-seq and DNase-seq [3]–[6]. These studies are mainly focused on identifying the specific genomic locations and DNA sequences associated with transcription factor binding and histone modifications. On the other hand are studies aimed at mapping various quantitative trait loci (QTL), such as gene expression levels (eQTLs) [7], DNA methylation (meQTLs) [8] and chromatin accessibility (dsQTLs) [9]. These studies are mainly focused on identifying specific genetic variants that functionally impact gene regulation. Cumulatively, binding map studies and QTL map studies have led to many insights into the principles and mechanisms of gene regulation [7], [10]–[12].

However, there are questions that neither mapping approach on its own is well equipped to address. One outstanding issue is the fraction of factor binding in the genome that is “functional”, which we define here to mean that disturbing the protein-DNA interaction leads to a measurable downstream effect on gene regulation. (Note that we do not concern ourselves with the question of whether the regulatory outcome and/or the interaction are evolving under natural selection). An experimental technique that could help address this issue is transcription factor knockdown. In knockdown experiments, the RNA interference pathway is employed to greatly reduce the expression level of a specific target gene by using small interfering RNAs (siRNAs). The cellular or organismal response to the knockdown can then be measured (e.g. [13]). Instead of measuring a cellular phenotype, one can collect RNA after the knockdown and measure global changes in gene expression patterns after specifically attenuating the expression level of a given factor.

Combining a TF knockdown approach with TF binding data can help us to distinguish functional binding from non-functional binding. This approach has previously been applied to the study of human TFs (e.g. [14]–[16]), although for the most part studies have only focused on the regulatory relationship of a single factor with its downstream targets. The FANTOM consortium previously knocked down 52 different transcription factors in the THP-1 cell line [17], an acute monocytic leukemia-derived cell line, and used a subset of these knockdowns to validate certain regulatory predictions based on binding motif enrichments [18]. However, the amount of transcription factor binding information available for the THP-1 cell line is limited (it is not a part of the ENCODE reference lines).

Many groups, including our own, have previously studied the regulatory architecture of gene expression in the model system of HapMap lymphoblastoid cell lines (LCLs) using both binding map strategies [3], [19], [20] and QTL mapping strategies [7], [9]. As a complement to that work, we sought to use knockdown experiments targeting transcription factors in a HapMap LCL to refine our understanding of the gene regulatory circuitry of the human genome. We integrated the results of the knockdown experiments with previous data on transcription factor binding to better characterize the regulatory targets of 59 different factors and to learn when a disruption in transcription factor binding is most likely to be associated with variation in the expression level of a nearby gene.

## Results

Our goal was to better characterize gene regulation by transcription factors (TFs). To do so, we measured the impact that knocking down the expression level of TFs and chromatin modifiers had on global gene expression levels in a single HapMap LCL (GM19238). As a first step, we used a high-throughput pipeline to screen siRNAs targeting 112 TFs for their efficiency in knocking down the target transcript (see Table S1 for a list of factors) to identify factors that were knocked down by at least 50%. We evaluated the knockdown efficiency using qPCR to measure transcript levels of the targeted gene in RNA samples extracted 48 hours after the siRNA transfection. Based on the qPCR results, we chose to focus on 59 TFs and chromatin modifiers, which were effectively knocked down using our approach (see Methods for specific details).

**Figure 1:**
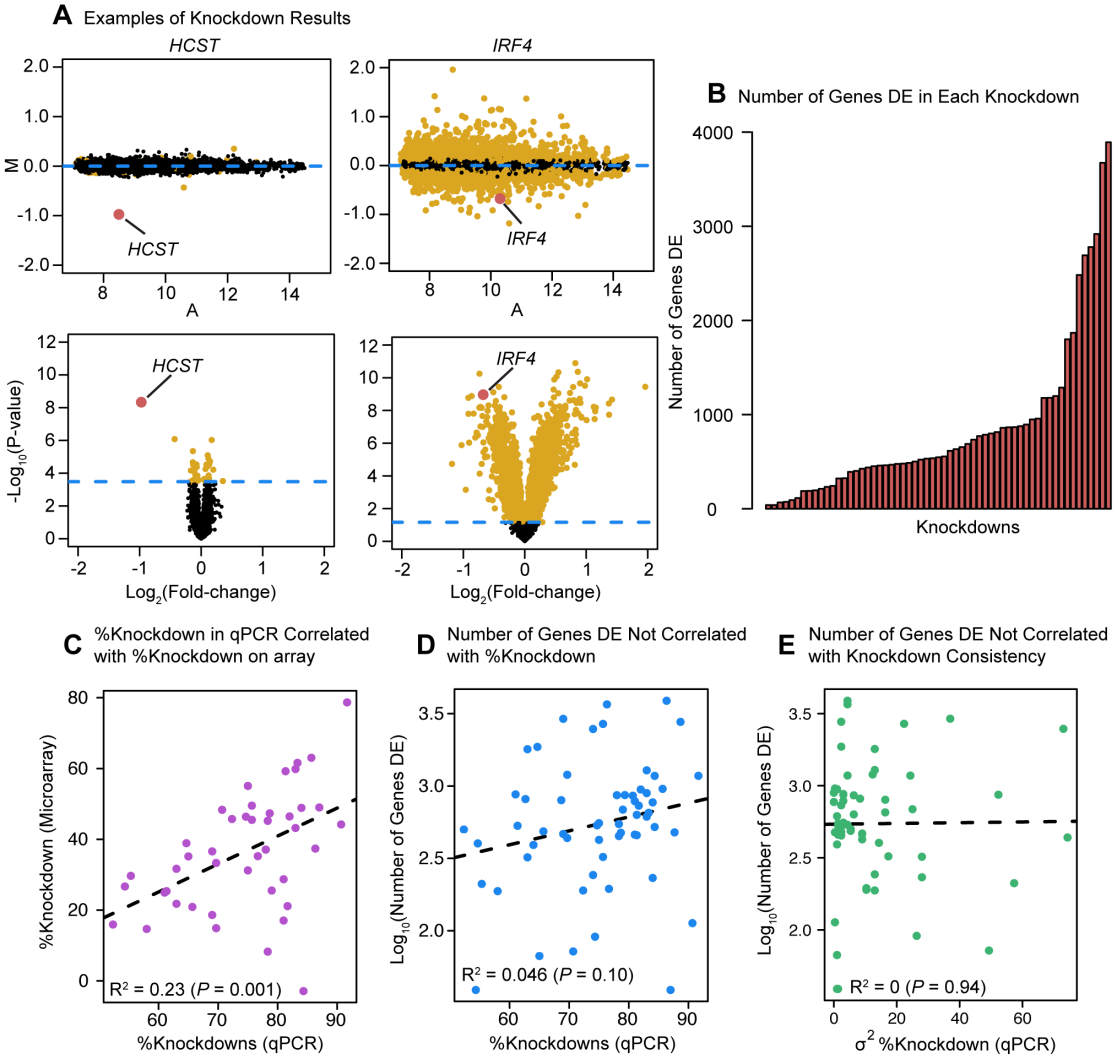
Differential expression analysis. (a) Examples of differential expression analysis results for the genes *HCST* and *IRF4*. The top two panels are ‘MA plots’ of the mean Log_2_(expression level) between the knockdown arrays and the controls for each gene (x-axis) to the Log_2_(Fold-Change) between the knockdowns and controls (y-axis). Differentially expressed genes at an FDR of 5% are plotted in yellow (points 50% larger). The gene targeted by the siRNA is highlighted in red. The bottom two panels are ‘volcano plots’ of the Log_2_(Fold-Change) between the knockdowns and controls (x-axis) to the P-value for differential expression (y-axis). The dashed line marks the 5% FDR threshold. Differentially expressed genes at an FDR of 5% are plotted in yellow (points 50% larger). The red dot marks the gene targeted by the siRNA. (b) Barplot of number of differentially expressed genes in each knockdown experiment. (c) Comparison of the knockdown level measured by qPCR (RNA sample collected 48 hours post-transfection) and the knockdown level measured by microarray. (d) Comparison of the level of knockdown of the transcription factor at 48 hrs (evaluated by qPCR; x-axis) and the number of genes differentially expressed in the knockdown experiment (y-axis). (e) Comparison of the variance in knockdown efficiency between replicates for each transcription factor (evaluated by qPCR; x-axis) and the number of differentially expressed genes in the knockdown experiment (y-axis).

We repeated the knockdown experiment for the 59 factors in triplicate, and collected RNA 72 hours after transfection for gene expression analysis using Illumina HT-12 microarrays. This time point was chosen to provide ample time for the transcript knockdown to impact the protein level of the targeted factor [21], [22]. All factors were knocked down in independent cultures of the same LCL. Gene expression levels following the knockdown were compared to expression data collected from six samples that were transfected with negative control siRNA. The expression data from all samples were normalized together using quantile normalization followed by batch correction using the RUV-2 method. We then performed several quality control analyses to confirm that the quality of the data was high, that there were no outlier samples, and that the normalization methods reduced the influence of confounders as much as possible (see Methods, Table S2 and Figures S1, S2, S3, S4, S5). Following these steps, we were able to consider expression data for 7,139–8,249 genes (depending on the TF knockdown experiment) that showed detectable expression on all of the knockdown arrays or all of the control arrays (Figure S6). In order to identify genes that were expressed at a significantly different level in the knockdown samples compared to the negative controls, we used likelihood-ratio tests within the framework of a fixed effect linear model (Figure 1, see Methods for details).

### Knockdown effect on global gene expression levels

Following normalization and quality control of the arrays, we identified genes that were differentially expressed between the three knockdown replicates of each factor and the six controls. Depending on the factor targeted, the knockdowns resulted in between 39 and 3,892 differentially expressed genes at an FDR of 5% (Figure 1B; see Table S3 for a summary of the results). The knockdown efficiency for the 59 factors ranged from 50% to 90% (based on qPCR; Table S1). The qPCR measurements of the knockdown level were significantly correlated with estimates of the TF expression levels based on the microarray data (*P* = 0.001; Figure 1C). Reassuringly, we did not observe a significant correlation between the knockdown efficiency of a given factor and the number of genes classified as differentially expressed following the knockdown experiment (this was true whether we estimated the knockdown effect based on qPCR (*P* = 0.10; Figure 1D) or microarray (*P* = 0.99; not shown) data. Nor did we observe a correlation between variance in qPCR-estimated knockdown efficiency (between replicates) and the number of genes differentially expressed (*P* = 0.94; Figure 1E). We noticed that the large variation in the number of differentially expressed genes extended even to knockdowns of factors from the same gene family. For example, knocking down *IRF4* (with a knockdown efficiency of 86%) resulted in 3,892 differentially expressed genes (including *IRF4*), while knocking down *IRF3* (with a knockdown efficiency of 91%), a paralog of *IRF4* [23], only significantly affected the expression of 113 genes (including *IRF3*).

**Figure 2:**
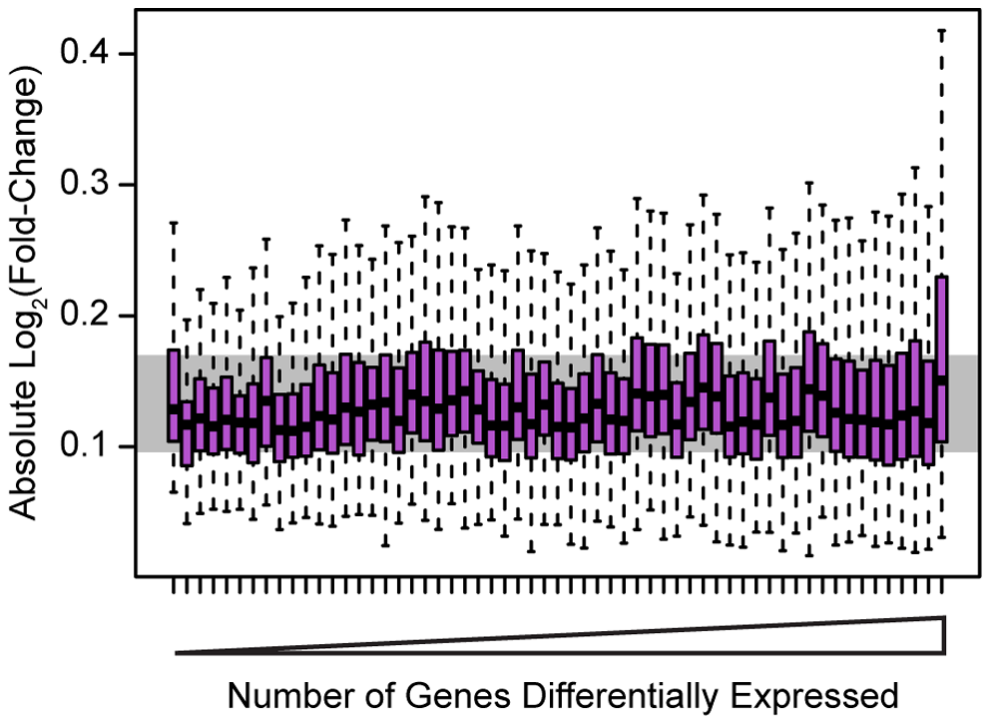
Effect sizes for differentially expressed genes. Boxplots of absolute Log_2_(fold-change) between knockdown arrays and control arrays for all genes identified as differentially expressed in each experiment. Outliers are not plotted. The gray bar indicates the interquartile range across all genes differentially expressed in all knockdowns. Boxplots are ordered by the number of genes differentially expressed in each experiment. Outliers were not plotted.

Because we knocked down 59 different factors in this experiment we were able to assess general patterns associated with the perturbation of transcription factors beyond merely the number of affected target genes. Globally, despite the range in the number of genes we identified as differentially expressed in each knockdown, the effect sizes of the differences in expression were relatively modest and consistent in magnitude across all knockdowns. The median effect size for genes classified as differentially expressed at an FDR of 5% in any knockdown was a 9.2% difference in expression level between the controls and the knockdown (Figure 2), while the median effect size for any individual knockdown experiment ranged between 8.1% and 11.0%.

To further evaluate the biological implications of our observations, we used the Gene Ontology (GO) [24] annotations to identify functional categories enriched among genes that were classified as differentially expressed following the knockdown experiments. In general, the differentially expressed genes tend to be annotated within pathways that fit well with what is already known about the biology of each of the 59 factors (Table S4). For example, differentially expressed genes following the knockdowns of both *IRF4* (3,892 genes differentially expressed) and *IRF9* (243 genes differentially expressed) are enriched for many immune response annotations. However, differentially expressed genes in the *IRF4* knockdown are enriched for both type I and II interferon signaling pathways, among other pathways, consistent with the known role of *IRF4* in immune responses [25]. Genes differentially expressed in the *IRF9* knockdown are enriched for type I interferon responses (among other pathways) but not type II responses, which is again consistent with the known biology [26]. As another example, knocking down *SREBF2* (1,286 genes differentially expressed), a key regulator of cholesterol homeostasis [27], results in changes in the expression of genes that are significantly enriched for cholesterol and sterol biosynthesis annotations. While not all factors exhibited striking enrichments for relevant functional categories and pathways, the overall picture is that perturbations of many of the factors primarily affected pathways consistent with their known biology.

### A combined analysis of factor binding and gene expression data

In order to assess functional TF binding, we next incorporated binding maps together with the knockdown expression data. In particular, we combined binding data based on DNase-seq footprints in 70 HapMap LCLs, reported by Degner et al. [9] (Table S5) and from ChIP-seq experiments in LCL GM12878, published by ENCODE [3]. We were thus able to obtain genome-wide binding maps for a total of 131 factors that were either directly targeted by an siRNA in our experiment (29 factors) or were differentially expressed in one of the knockdown experiments (see Methods for more details). We classified a gene as a bound target of a particular factor when binding of that factor was inferred within 10kb of the transcription start site (TSS) of the target gene. Using this approach, we found that the 131 TFs were bound in proximity to a median of 1,922 genes per factor (range 11 to 7,053 target genes; Figure S7A; only the 8,872 genes expressed in at least one knockdown experiment were considered for this analysis). Target genes were bound by a median of 34 different factors (range 0 to 96; Figure S7B; only 288 genes expressed in our experiments were not classified as bound targets of any of the 131 factors considered).

We considered binding of a factor to be functional if the target gene was differentially expressed after perturbing the expression level the bound transcription factor. We then asked about the concordance between the transcription factor binding data and the knockdown expression data. Specifically, we studied the extent to which differences in gene expression levels following the knockdowns might be predicted by binding of the transcription factors within the putative regulatory regions of the responsive genes. Likewise, we asked what proportion of putative target (bound) genes of a given TF were also differentially expressed following the knockdown of the factor.

We performed this analysis in two stages. First, we only considered binding data for the specific TF that was knocked down in each experiment (binding data was available for 29 different factors). In general, we found that the number of differentially expressed genes following the knockdowns was positively correlated with the number of bound target genes by these 29 factors (Spearman's ρ = 0.45; permutation *P* = 0.015). For 12 of the 29 knockdowns, we observed significant overlaps between binding and differential expression (Fisher's exact test; *P*<0.05). We also found that between 3.4–75.9% (median = 32.3%) of differentially expressed genes were bound by the TF in a given knockdown (mean relative enrichment = 1.08). Perhaps somewhat less expected, we found that between 46.4% and 99.1% (median = 88.9%) of the binding was apparently not functional, namely it was not associated with changes in gene expression levels. This observation is robust with respect to the size of the window we used to classify genes as bound by a factor (range 1–20 kb from the TSS; Table S6). It is also consistent with our previous findings that most DNase-I sensitive QTLs are not also classified as eQTLs [9].

**Figure 3:**
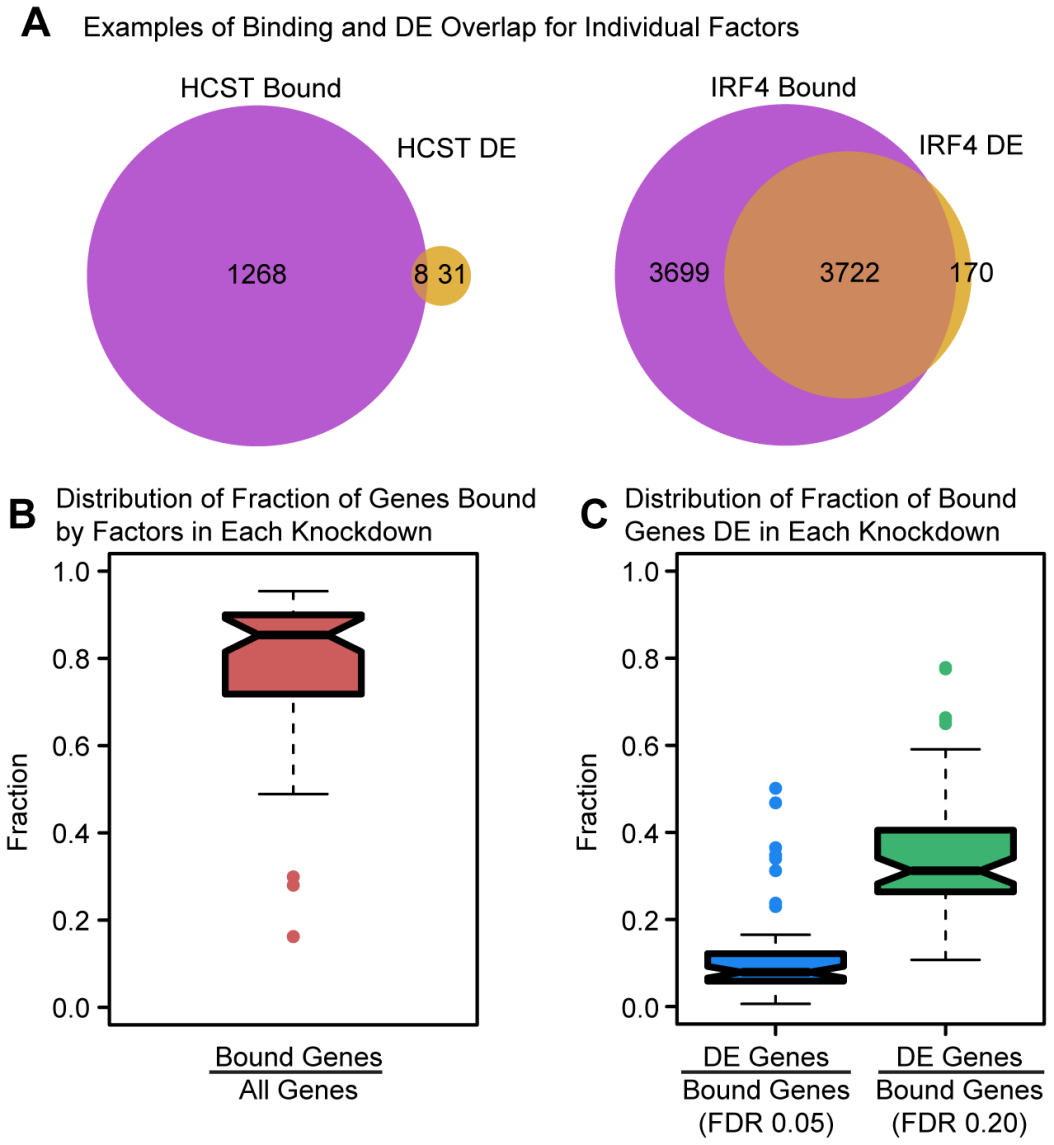
Intersecting binding data and expression data for each knockdown. (a) Example Venn diagrams showing the overlap of binding and differential expression for the knockdowns of *HCST* and *IRF4* (the same genes as in Figure 1). (b) Boxplot summarizing the distribution of the fraction of all expressed genes that are bound by the targeted gene or downstream factors. (c) Boxplot summarizing the distribution of the fraction of bound genes that are classified as differentially expressed, using an FDR of either 5% or 20%.

We next considered the expression data in the context of the binding data for both the knocked down TFs and any other TF whose expression level was indirectly affected by the knockdown. We again examined the overlap between binding and differential expression (Figure 3). Considering only the expressed genes in each experiment, the fraction of genes bound (by any TF whose expression has changed) ranged between 16.2% and 95.3% with a median of 85.4% (Figure 3B). However, the fraction of bound genes that were also differentially expressed in a given experiment was generally quite low (median = 7.9%; Figure 3C; mean relative enrichment = 1.02), with significant (*P*<0.05) overlap between bound and differentially expressed genes seen for only 13 of the 59 knockdown experiments. Even if we relaxed the statistical threshold with which we classify genes as differentially expressed four-fold (to an FDR of 20%), a majority of bound genes still failed to show significant evidence of differential expression (median = 68.8%; Figure 3C; mean relative enrichment = 1.01). The discrepancy in the number of genes bound by a particular factor (or the TFs it regulates) and the number of differentially expressed genes in a knockdown experiment begged the question of whether any characteristics of factor binding might distinguish functionally bound target genes. In order to address this question, we examined a variety of features.

### Functional factor binding is enriched in enhancer chromatin states

First, focusing only on the binding sites classified using the DNase-seq data (which were assigned to a specific instance of the binding motif, unlike the ChIP data), we examined sequence features that might distinguish functional binding. In particular, we considered whether binding at conserved sites was more likely to be functional (estimating conservation by using PhastCons 46 way placental scores [28]) and we also considered whether binding sites that better matched the known PWM for the factor were more likely to be functional. Interestingly, we did not observe a significant shift in the conservation of functional binding sites (Wilcoxon rank sum *P* = 0.34), but we did observe that binding around differentially expressed genes occurred at sites that were significantly better matches to the canonical binding motif (*P*<10^−8^), although the absolute difference in PWM score was very small.

**Figure 4:**
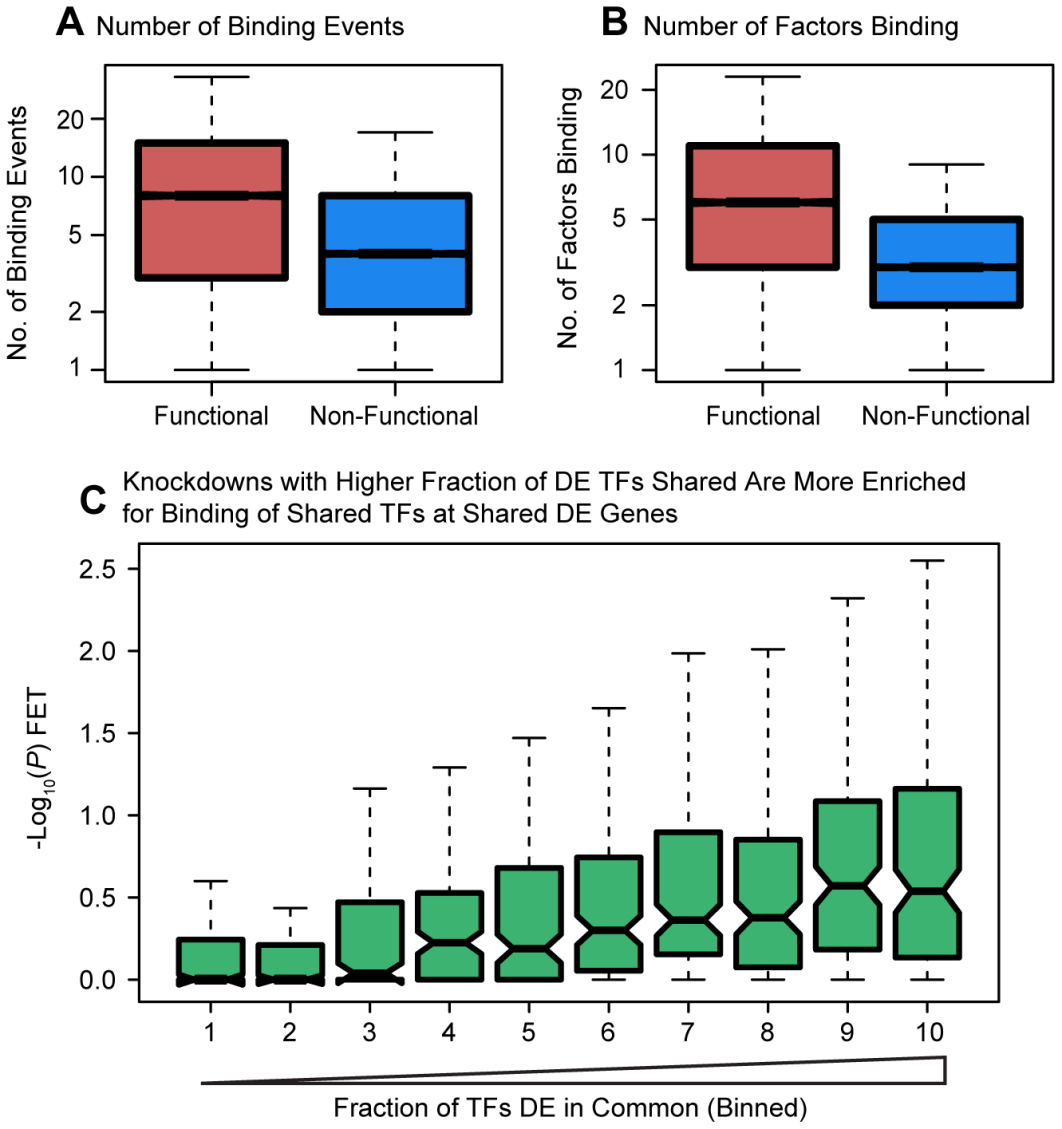
Degree of binding correlated with function. Boxplots comparing (a) the number of sites bound, and (b) the number of differentially expressed transcription factors binding events near functionally or non-functionally bound genes. We considered binding for siRNA-targeted factor and any factor differentially expressed in the knockdown. (c) Focusing only on genes differentially expressed in common between each pairwise set of knockdowns we tested for enrichments of functional binding (y-axis). Pairwise comparisons between knockdown experiments were binned by the fraction of differentially expressed transcription factors in common between the two experiments. For these boxplots, outliers were not plotted.

Next, considering bound targets determined from either the ChIP-seq or DNase-seq data, we observed that differentially expressed genes were associated with both a higher number of binding events for the relevant factors within 10 kb of the TSS (*P*<10^−16^; Figure 4A) as well as with a larger number of different binding factors (considering the siRNA-targeted factor and any TFs that were DE in the knockdown; *P*<10^−16^; Figure 4B). We hoped to distinguish between coordinated co-regulation of the factors and generally higher levels of binding nearby differentially expressed genes. To do so, we asked whether the genes differentially expressed in common between any two knockdown experiments were more likely to be co-occupied by the same transcription factors (considering only transcription factors whose expression was affected by the knockdown). Binning all pairwise comparisons between knockdown experiments based on the fraction of differentially expressed transcription factors in common, we observed that enrichment for functional co-occupancy increased proportionally to the fraction of TFs in common (Figure 4C). This suggests that co-regulation is at least partially responsible for the increased numbers of factors binding near differentially expressed genes.

**Figure 5:**
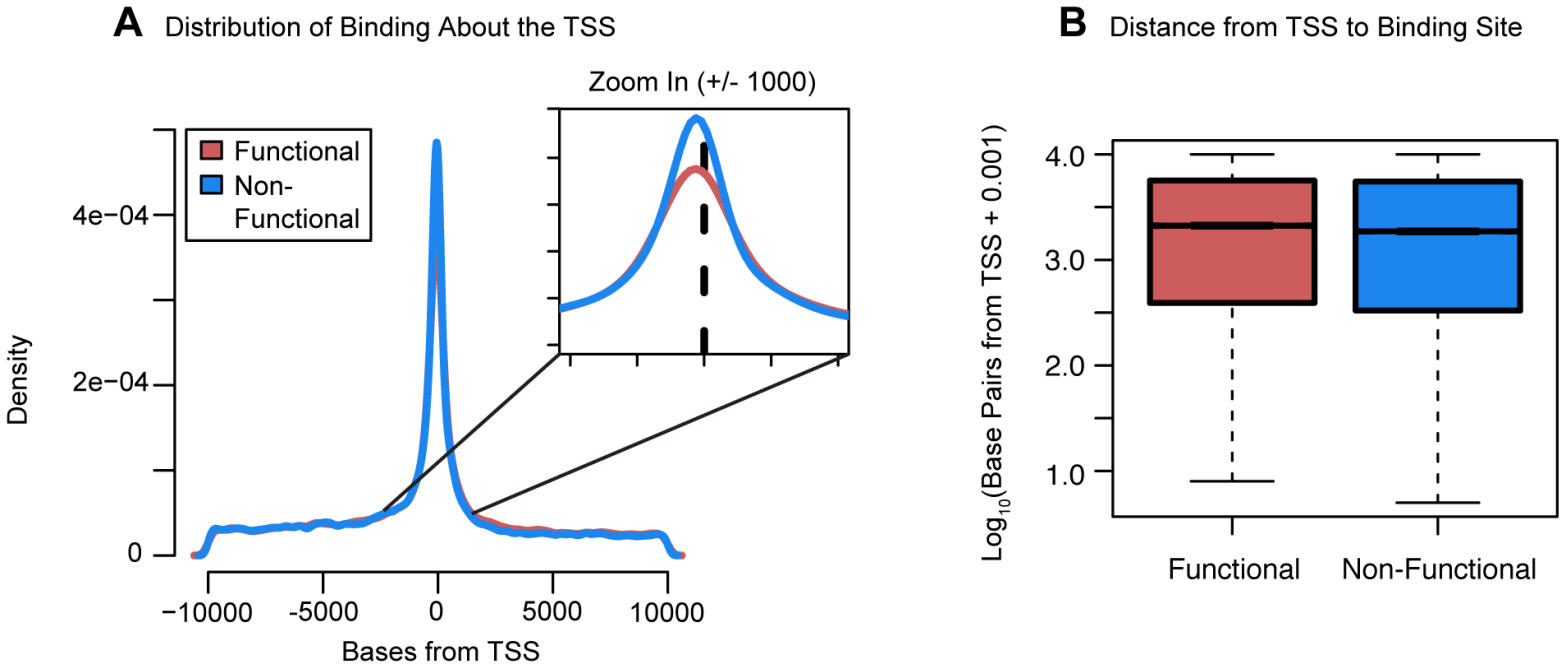
Distribution of functional binding about the TSS. (a) A density plot of the distribution of bound sites within 10 kb of the TSS for both functional and non-functional genes. Inset is a zoom-in of the region +/−1 kb from the TSS (b) Boxplots comparing the distances from the TSS to the binding sites for functionally bound genes and non-functionally bound genes. For the boxplots, 0.001 was added before log10 transforming the distances and outliers were not plotted.

We proceeded by examining the distribution of binding about the TSS. Most factor binding was concentrated near the TSS whether or not the genes were classified as differentially expressed (Figure 5A). However, surprisingly, the distance from the TSS to the binding sites was significantly longer for differentially expressed genes (*P*<10^−16^; Fig. 5B). We then investigated the distribution of factor binding across various chromatin states, as defined by Ernst et al. [11]. This dataset lists regions of the genome that have been assigned to different activity states based on ChIP-seq data for various histone modifications and *CTCF* binding. For each knockdown, we separated binding events by the genomic state in which they occurred and then tested whether binding in that state was enriched around differentially expressed genes. After correcting for multiple testing, 19 knockdowns showed significant enrichment for binding in “strong enhancers” around genes that were differentially expressed and four knockdowns had significant enrichments for “weak enhancers”. Further, eight knockdowns showed significant depletion of binding in “active promoters” of genes that were differentially expressed and six knockdowns had significant depletions for “transcription elongation”.

### The direction of expression change

**Figure 6:**
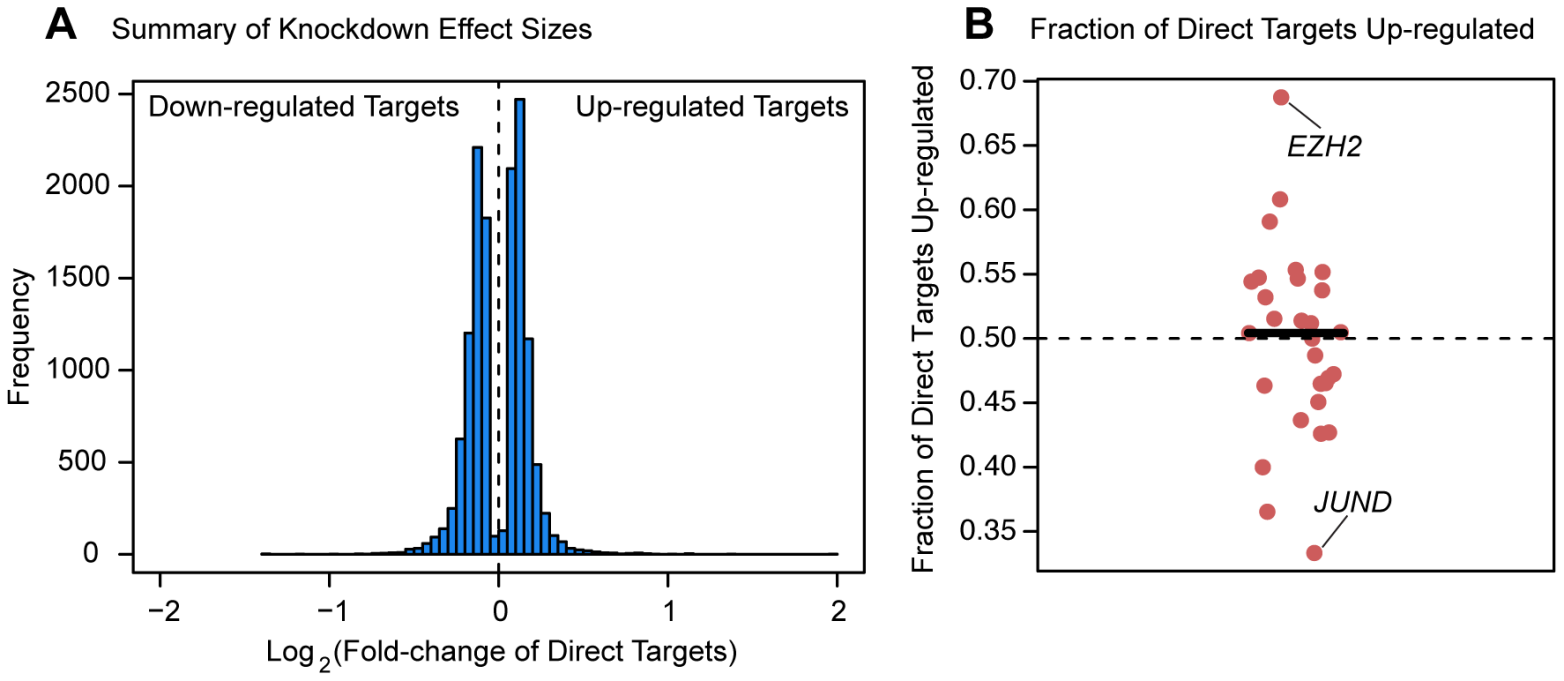
Magnitude and direction of differential expression after knockdown. (a) Density plot of all Log_2_(fold-changes) between the knockdown arrays and controls for genes that are differentially expressed at 5% FDR in one of the knockdown experiments as well as bound by the targeted transcription factor. (b) Plot of the fraction of differentially expressed putative direct targets that were up-regulated in each of the knockdown experiments.

Finally, we asked whether the factors tended to have a consistent effect (either up- or down-regulation) on the expression levels of genes they purportedly regulated. Perhaps surprisingly, all factors we tested are associated with both up- and down-regulation of downstream targets (Figure 6). A slight majority of downstream target genes were expressed at higher levels following the knockdown for 15 of the 29 factors for which we had binding information (Figure 6B). The factor that is associated with the largest fraction (68.8%) of up-regulated target genes following the knockdown is *EZH2*, the enzymatic component of the Polycomb group complex. On the other end of the spectrum was *JUND*, a member of the AP-1 complex, for which 66.7% of differentially expressed targets were down-regulated following the knockdown. The remaining 27 factors (with a median of 170 direct targets) all show a more even balance between up- and down-regulated targets. These trends are consistent when we considered all genes that were differentially expressed following a knockdown (not just the genes that were also bound by the knocked down factor). We observed that an average of 51.9% of downstream differentially expressed genes had elevated expression levels following knockdown of the transcription factors. Furthermore, for 39 of the experiments a slight majority of differentially expressed genes following knockdown of the factor were up-regulated, while in only 17 of the experiments were the majority of differentially expressed genes down-regulated following the knockdown.

## Discussion

The question of the distinction between functional and non-functional DNA in the human genome has attracted considerable attention recently. Some have argued that any base that comes into contact with a protein or RNA molecule at some point in its lifespan should be considered functional [3], which is an extremely inclusive definition. Conversely, others have argued that only the bases of the genome that evolve under natural selection pressure and can be clearly ascribed to a particular phenotype should be considered functional [29]. We agree that functional interpretation makes more sense in an evolutionary context, but we also recognize that our ability to identify the signatures of natural selection (especially positive selection) is limited. Instead, we propose an experimentally tractable definition of functional interactions between transcription factors and DNA, which allowed us to make several novel observations regarding the nuanced regulatory logic of the human genome.

### Characterizing functional transcription factor binding

Nearly all expressed genes in the LCL we worked with are bound within 10 kb of their TSS by at least one of the 131 TFs for which we were able to obtain binding data. Yet, the regulation of the vast majority of target genes is not affected by perturbations to the expression levels of the TFs. Our observations suggest that it may ultimately be possible to predict functional transcription factor binding based on the biological context, yet since bound genes are only modestly enriched among those that are differentially expressed, an effective classifier may be difficult to develop. In that context, several of the associations we observed might seem counterintuitive from a purely biochemical perspective but are consistent with our definition of functional TF binding as participating in gene regulation. In particular, from a biochemical perspective, binding at stronger motifs might be expected to be less affected by a decrease in factor concentration in the cell (following the knockdown) and regulatory regions with more binding sites and a larger number of bound factors might be expected to be less influenced by the perturbation of one single factor. Yet, we observed the opposite patterns: Functional binding is associated with stronger binding motifs and greater levels of factor binding near differentially expressed genes. Viewed from an evolutionary rather than purely biochemical perspective, these observations are quite in tune with expectations. In other words, genomic regions of functional importance evolve to ensure factor binding. This can be accomplished by selection for different properties, including increased affinity of the binding site and more cooperative binding.

Our results also indicate that binding in the context of certain chromatin states was more likely to be functional. For 19 of the 56 factors that we knocked down and for which we were able to obtain binding data (on either direct or indirect bound targets), there was a significant enrichment of binding in “strong enhancers” near differentially expressed genes, which is consistent with our observations that functional binding occurs further from the TSS (namely, not in promoter regions). While further experiments are required before we can put forward a more concrete explanation, these observations suggest that binding at active promoters may be buffered against acute changes in transcription factor concentration. This may also explain why most of the effect sizes associated with differences in gene expression levels following the knockdowns were relatively modest. While there is compelling evidence for our inferences, the current chromatin functional annotations do not fully explain the regulatory effects of the knockdown experiments. For example, the enrichments for binding in “strong enhancer” regions of the genome range from 7.2% to 50.1% (median = 19.2%), much beyond what is expected by chance alone, but far from accounting for all functional binding.

Previous studies in model organisms suggested redundancy as a partial explanation for the common observation of non-functional TF binding. For example, Gitter et al. examined similar themes using gene expression data from yeast in which transcription factors had been knocked out [30]. They found that factors without identifiable paralogs were more likely to have a regulatory affect on bound targets than factors with identifiable paralogs, which can potentially have overlapping functions. We considered a similar explanation for our observations. To do so, we used Ensembl's definition of paralogs [31] to stratify the 29 factors that we knocked down, and for which we also had direct binding data, by the number of identifiable paralogs each had (range 1–5). We found no correlation between the number of paralogs and the fraction of bound targets that were differentially expressed. We also did not observe a significant correlation when we considered whether the percent identity of the closest paralog might be predicative of the fraction of bound genes that were differentially expressed following the knockdown (Figure S8).

### Evaluating the impact of transcription factor binding on direct targets

In addition to considering the distinguishing characteristics of functional binding, we also examined the direction of effect that perturbing a transcription factor had on the expression level of its direct targets. We specifically addressed whether knocking down a particular factor tended to drive expression of its putatively direct (namely, bound) targets up or down, which can be used to infer that the factor represses or activates the target, respectively. Transcription factors have traditionally been thought of primarily as activators [11], [32], [33], and previous work from our group is consistent with that notion [9]. Surprisingly, the most straightforward inference from the present study is that many of the factors function as repressors at least as often as they function as activators. For example, we inferred that *EZH2* had a negative regulatory relationship with the largest fraction of direct targets (68.8%), while *JUND* seemed to have a positive regulatory relationship with the largest fraction of direct targets (66.7%). These particular observations seem consistent with the known role of *EZH2* as the active member of the Polycomb group complex PC2 [34] and the biochemical characterization of the AP-1 complex (of which *JUND* is a component) as a transactivator [35]. More generally, however, our results, combined with the previous work from our group and others (e.g. [9], [11]) make for a complicated view of the role of transcription factors in gene regulation as it seems difficult to reconcile the inference from previous work that many transcription factors should primarily act as activators with the results presented here.

One somewhat complicated hypothesis, which nevertheless can resolve the apparent discrepancy, is that the “repressive” effects we observe for known activators may be at sites in which the activator is acting as a weak enhancer of transcription and that reducing the cellular concentration of the factor releases the regulatory region to binding by an alternative, stronger activator. We believe that this may alleviate the apparently contradictory interpretations of transcription factor activity, although additional work on this topic is needed.

### Possible caveats and future studies

There are important caveats that should be kept in mind when interpreting our results. Care must be taken in interpreting the lack of evidence for gene expression differences in our system as previous studies have suggested that the specific effects of knockdowns may be difficult to detect because of redundancy in gene regulatory mechanisms [36], [37]. In addition, our definition of target genes, which is based on genomic proximity, is certain to be somewhat noisy. For some genes, we are undoubtedly assigning irrelevant binding while for others we are missing true regulatory interactions that may occur further than 10 kb from the transcription start site. Moreover, we only considered binding and expression in one set of conditions (one cell type at one timepoint). Some binding events may require co-factors that are only present after specific stimuli in order to become functional or may otherwise be functional in other contexts. Because of all of this, we are likely underestimating the fraction of bound genes that are functionally regulated. We have taken measures to control for this as much as possible. First, integrating our results with binding data allows us to focus our analysis on the most likely direct targets. Second, the consistency of our results across a variety of factors (including factors with very different numbers of differentially expressed genes following the knockdown), suggests that technical explanations for our observations are unlikely.

To more explicitly address the effect that our proximity-based definition of target genes might have on our analyses, we reanalyzed the overlap between factor binding and differential expression following the knockdowns using an independent, empirically determined set of target genes. Specifically, Thurman et al. used correlations in DNase hypersensitivity between intergenic hypersensitive sites and promoter hypersensitive sites across diverse tissues in order to assign intergenic regulatory regions to specific genes, independently of proximity to a particular promoter [38]. We thus performed an alternative analysis in which we assigned binding events to genes based on the classification of Thurman et al. We then considered the overlap between binding and differential expression in this new dataset. The results were largely consistent with our proximity-based observations. A median of 9.5% of genes that were bound by a factor were also differentially expressed following the knockdown of that factor (compared to 11.1% when the assignment of binding sites to genes is based on proximity). From the opposite perspective, a median of 28.0% of differentially expressed genes were bound by that factor (compared to 32.3% for the proximity-based definition). The results of this analysis are summarized in Table S7.

Another practical limitation of our study design is the choice of microarrays instead of a more advanced technology such as RNA-seq. As a result of this choice, the expression of only the genes represented by probes on the arrays could be measured. In addition, alternative splicing events and novel transcription were not measured in our experiments. Furthermore, microarrays are known to have sensitivity issues at the extreme ranges of expression levels (although this will only affect a small subset of transcripts) [39]. Because of this, our results should not be considered a comprehensive census of regulatory events in the human genome. Instead, we adopted a gene-centric approach, focusing only on binding events near the genes for which we could measure expression to learn some of the principles of functional transcription factor binding.

In light of our observations a reassessment of our estimates of binding may be warranted. In particular, because functional binding is skewed away from promoters (our system is apparently not well-suited to observe functional promoter binding, perhaps because of protection by large protein complexes), a more conservative estimate of the fraction of binding that is indeed functional would not consider data within the promoter. Importantly, excluding the putative promoter region from our analysis (i.e. only considering a window >1 kb from the TSS and <10 kb from the TSS) does not change our conclusions. Considering this smaller window, a median of 67.0% of expressed genes are still classified as bound by either the knocked down transcription factor or a downstream factors that is differentially expressed in each experiment, yet a median of only 8.1% of the bound genes are also differentially expressed after the knockdowns.

Further work in this field is clearly justified. Much of what distinguishes functional binding (as we define it) has yet to be explained. Furthermore, we are unable to explain much of the differential expression observed in our experiments by the presence of least one relevant binding event. This may not be altogether surprising, as we are only considering binding in a limited window around the transcription start site. To address these issues, more factors should be perturbed to further evaluate the robustness of our results and to add insight. Together, such studies will help us develop a more sophisticated understanding of functional transcription factor binding in particular, the gene regulatory logic more generally.

## Methods

### Cell culture and siRNA transfections

The cell line (GM19238) was cultured at 5% CO_2_ and 37°C in RPMI 1640 medium supplemented with 2 mM L-glutamine and 15% fetal bovine serum, per Coriell's recommendations (http://ccr.coriell.org/Sections/Support/Global/Lymphoblastoid.aspx?PgId=213). The medium was also supplemented with 100 IU/ml penicillin and 100 µg/ml streptomycin. Cells were counted and split three times a week to 350,000 cells/ml. Transfections were performed with the Lonza 96-well nucleofector system, using transfection solution SF and transfection program DN-100. On-Target SmartPool siRNAs from Dharmacon were used to knockdown target genes. For each transfection, one million cells were transfected with 50 pmol siRNA. After transfection, cells were plated in 1 ml of medium in 96-well plates and incubated for 48. At the 48 hour time point, 500 µl of cell culture were removed for RNA extraction (for qPCR), 100 µl of fresh medium was added to each well and plates were incubated for an additional 24 hours. At the 72 hour time point, the remaining culture was pelleted for RNA extraction (for array hybridization). RNA from all timepoints was extracted using the RNeay Plus 96 kit (Qiagen). The control siRNAs consisted of a pool of four siRNAs specifically designed not to target any human or mouse gene. In addition to two replicates of the negative control pool, we also transfected each of the negative control siRNAs independently for a total of six negative control samples. We learned from an initial pilot experiment that there were strong batch effects between rounds of transfection and so we included negative control transfection in parallel with all three batches of transfection.

The transfections were conducted in two phases. For the first phase, we screened siRNAs for their knockdown efficiency under our experimental conditions by transfecting cells and extracting RNA 48 hours later. qPCR was performed with SYBR Green and custom primers (Table S1). Knockdown efficiency was assessed relative to a sample transfected with the negative control siRNA pool. We used a relative quantification approach, referencing the *POLR2C* control gene and using the DART-PCR method [40] to determine PCR efficiency. For the second phase, those siRNA transfections resulting in ≥50% knockdown (arbitrary cutoff) were transfected again and RNA was extracted at both 48 hours (to confirm the knockdown level by qPCR) and 72 hours (for hybridization to microarrays). Two genes (*NFYC* and *ZHX2*) were not knocked down by 50% in the screen but were included in the final transfection where they did reach 50% (in all three replicates) and were therefore included in the microarray experiment. Additionally, 16 factors that passed the screen were not knocked down by 50% at 48 hours in all three replicates in the final transfection and were not included in the microarray experiment (Table S1). The quality and concentration of all RNA samples were measured using the Agilent 2100 Bioanalyzer.

### Transcription factors and chromatin modifiers targeted by siRNA

*ARNTL2*, *BATF*, *BCL3*, *CEBPG*, *CEBPZ*, *CLOCK*, *CREBBP*, *DIP2B*, *E2F1*, *E2F4*, *E2F6*, *EP300*, *ESRRA*, *EZH2*, *FOXA3*, *GTF2B*, *HCST*, *HOXB7*, *IKZF3*, *IRF3*, *IRF4*, *IRF5*, *IRF7*, *IRF8*, *IRF9*, *JUND*, *KLF13*, *LCORL*, *NFE2L1*, *NFKB2*, *NFX1*, *NFYC*, *NR1D2*, *NR2F6*, *NR3C1*, *PAX5*, *POU2F1*, *POU2F2*, *RAD21*, *RDBP*, *RELA*, *RELB*, *RXRA*, *SKIL*, *SP1*, *SP3*, *SREBF2*, *STAT2*, *STAT6*, *TAF1*, *TCF12*, *TFDP1*, *TFDP2*, *TFE3*, *USF1*, *WHSC1*, *YY1*, *ZBTB38*, *ZHX2*.

### Gene expression arrays and normalization

Samples were hybridized to Illumina HT-12 v4R2 arrays in two batches. For the first batch, 69 samples (150 ng of total RNA), representing 21 knockdowns and one set of controls, were sent to the UCLA Southern California Genotyping Consortium where the RNA was converted to cRNA and then hybridized to arrays using the standard protocol. For the second batch, 132 samples (1 µg of total RNA), representing 40 knockdowns and two sets of controls, were sent to the University of Chicago Functional Genomics Core where the RNA was converted to cRNA and then hybridized to the arrays using the standard protocol. Both cores returned raw probe intensities to us. The batch aspect of the study design is clearly less than optimal, but it was a result of necessary practical considerations. As mentioned above, we included a full set of negative controls with each batch. We also accounted for batch effects explicitly in our analysis (see below).

Before processing the arrays, we determined the set of usable probes. To do this, we first mapped probes to the hg19 reference genome using the BWA alignment program [41] and only retained probes that mapped perfectly to the genome (probes spanning introns were discarded). We then removed probes that mapped perfectly to a single site in the genome and also mapped to a second site allowing for one mismatch. As GM19238 was derived from a female donor, we excluded all probes for Y chromosome genes. We then removed probes that contained a SNP for which GM19238 was heterozygous based on 1000 Genomes data [42] (http://ftp.1000genomes.ebi.ac.uk/vol1/ftp/pilot_data/paper_data_sets/a_map_of_human_variation/trio/snps/) to avoid detecting spurious results from an interaction between allele specific expression and probe effects influencing hybridization intensities. After this series of exclusions, for each gene that was assayed by more than one remaining probe, we chose the 3′-most probe to represent the gene. We then excluded probes for genes that were not expressed (detection *P*<0.01) in the knockdown triplicates or the full set of controls (18 arrays) for each experiment.

We log-transformed and quantile normalized the data from all arrays together using the ‘lumi’ Bioconductor package [43], [44] in the R statistical environment. Our initial analysis indicated that this was not sufficient to correct for all of the batch effects present between different rounds of transfection (Figure S5), so we also used the RUV-2 method [45] to further adjust the data. To do so, we defined a set of genes that should not be differentially expressed under any conditions in our experiment as the basis for the RUV-2 correction. While the choice of unaffected genes might not be straightforward in all experimental settings, the fact that we had 59 different knockdown experiments (and controls) across three rounds of transfections provided an opportunity to define a reasonable set of control probes. Specifically, we created a list of the 2,000 least variable probes (considering only probes for genes that passed our expression threshold) for each of the batches of arrays separately. We then intersected the three lists to obtain a high confidence list of 787 invariant genes. These are the genes with the least evidence of difference in expression levels in any knockdown experiment.

We also needed to specify the number of components to regress out. Again, this choice may not always be straightforward, however, we had two knockdown experiments (*IRF5* and *SP1*) that were each repeated across different batches. We thus maximized the correlation between these experiments in deciding on the number of components to remove. We found that removing eight components resulted in zero genes identified as differentially expressed (at an FDR of 5%) between replicate experiments (or the control arrays by themselves, across batches) and had the maximum number of differentially expressed genes replicated between the two experiments for both *IRF5* and *SP1* (Figure S2; these models were fit with the ‘limma’ package [46]). Lastly, we averaged each of the negative control knockdowns across each of the time points in order to have a single set of controls with which to compare each of the knockdowns. In addition to examining heatmaps for the arrays (Figures S1, S3), the quality of each array was assessed by relative log expression (‘RLE’) plots (Figure S4; [47], [48]) and PCA (Figure S5, Table S2). For the remainder of the analyses, we only used the *IRF5* and *SP1* experiment that had the greater knockdown efficiency.

We note that the top principle components of the adjusted data are still correlated with the microarray chip (Table S2), but we decided that further correction was not helpful for several reasons. First, the amount of variance captured by any one component is quite low after RUV-2 adjustment (Table S2). Second, our analysis indicated that removing additional components with RUV-2 reduced the correlations between replicate knockdown experiments (Figure S2). Third, we randomized samples across chips so as to avoid the introduction of a bias in our results based on such an effect.

### Determining differentially expressed genes

In order to identify differentially expressed genes in each knockdown, we compared the expression level of each gene on the three knockdown arrays to its expression level on the six control arrays. We used a likelihood-ratio test within the framework of a fixed-effect linear model:
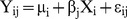
Here, Y_ij_ is the expression level of gene i on array j. μ_i_ is the mean expression level for gene i. β_j_ is the status of array j (either “knockdown” or “control”) and X_i_ is the knockdown effect for gene i. ε_ij_ is the error term for gene i on array j. For each gene, we compared a model with a β_j_X_i_ term to the nested model with no β_j_X_i_ term using a likelihood ratio test to determine which model fit the data better. To adjust for multiple testing within each knockdown experiment, we calculated the q-value for each gene using the Storey and Tibshirani method [49] as provided in the ‘qvalues’ R package.

### Gene ontology analysis

For each knockdown, we also assessed whether there were any Gene Ontology categories (either from the “MF” or “BP” categories) that were overrepresented among the differentially expressed genes. To do so, we used the ‘topGO’ package and the ‘org.Hs.eg’ database in R. For each knockdown, we tested both “MF” and “BP” categories, combined the results, and filtered out any categories without a single gene differentially expressed. P-values were adjusted for multiple testing using the ‘BH’ method in the ‘p.adjust’ function in R.

### Combining with other genomic datasets

After identifying differentially expressed genes, we intersected the gene expression data with factor binding data from both DNase-seq experiments and ChIP-seq experiments. We considered binding data within a fixed window around the TSS of each gene. To determine a single TSS for each gene, we used the ENCODE CAGE data downloaded from UCSC [3], [50]. For each gene, we chose the TSS with the highest CAGE score as the reference TSS for that gene unless there was a tie between multiple TSSs, in which case we used the midpoint between these TSSs as the reference TSS. The DNase data was from a previous study conducted in our lab [9]. Binding was determined using the Centipede algorithm [4] on DNase-seq data from 70 Yoruba HapMap cell lines. For each factor expressed in our experiments, we classified all sites with a Centipede posterior probability greater than 0.95 as bound. The binding was originally mapped to the hg18 reference genome, so we used liftOver (http://hgdownload.cse.ucsc.edu/admin/exe/) to convert the coordinates to h19.

For the ChIP data, we downloaded all ENCODE ChIP-seq data for GM12878 called using the SPP peak caller, except for the *POL2* datasets and an *NFKB* dataset that was collected following tumor necrosis factor-α stimulation [3] (http://ftp.ebi.ac.uk/pub/databases/ensembl/encode/integration_data_jan2011/byDataType/peaks/jan2011/spp/optimal/hub/). For each gene that we were able to link to a ChIP dataset or a TRANSFAC binding motif [51], we combined all binding records and then considered the union of that set as the binding profile for the factor. After obtaining the union set, we calculated the midpoint for each discrete binding record and used the midpoints as the estimated binding location in all subsequent analyses.

Using this approach, we obtained binding data for 201 factors; 138 of these factors were represented by a usable probe on the array and were expressed in at least one of the knockdown experiments. 131 of the factors were differentially expressed in at least one knockdown experiment. For DNase-based binding sites, we also evaluated the PhastCons alignment score [28]. PhastCons 46 way placental wig files were downloaded from UCSC and the average score for each DNase-seq binding site was calculated. For all of the comparisons between functional and non-functional binding, we used the Wilcoxon rank sum test to assess differences.

We also downloaded the Ernst chromatin states [11] from UCSC. This file contained 15 different chromatin states, including two separate categories each for “strong enhancer”, “weak enhancer”, and “repetitive”. We combined each of the replicate states into a single category so that we ended up with 12 distinct chromatin states. To identify which state the binding occurred in, we intersected the binding record midpoints with the chromatin states. We calculated the enrichment or depletion of functional binding in specific chromatin states using a Fisher's Exact Test.

To test whether the number of paralogs or the degree of similarity with the closest paralog for each transcription factor knocked down might influence the number of genes differentially expressed in our experiments, we obtained definitions of paralogy and the calculations of percent identity for 29 different factors from Ensembl's BioMart (http://useast.ensembl.org/biomart/martview/) [31]. We used genome build GRCh37.p13. For each gene, we counted the number of paralogs classified as a “within_species_paralog”. After selecting only genes considered a “within_species_paralog”, we also assigned the maximum percent identity as the closest paralog. To evaluate the effect that an independent assignment of target genes to regulatory regions might have on our analyses, we used the definition of target genes defined by Thurman et al. (ftp://ftp.ebi.ac.uk/pub/databases/ensembl/encode/integration_data_jan2011/byDataType/openchrom/jan2011/dhs_gene_connectivity/genomewideCorrs_above0.7_promoterPlusMinus500kb_withGeneNames_32celltypeCategories.bed8.gz), which use correlations in DNase hypersensitivity between distal and proximal regulatory regions across different cell types to link distal elements to putative target genes [38]. We intersected the midpoints of our called binding events (defined above) with these regulatory elements in order to assign our binding events to specific target genes and then re-analyzed the overlap between binding and differential expression in our experiments.

All analyses were performed with a combination of BedTools [52], [53] and BEDOPS [54] commands, along with custom Python and R scripts.

### Data availability

The knockdown data have been deposited in NCBI's Gene Expression Omnibus ([55]; accessible through GEO Series accession number GSE50588 at http://www.ncbi.nlm.nih.gov/geo/query/acc.cgi?acc=GSE50588)

## Supporting Information

Figure S1 Heatmap of microarray correlations. A heatmap showing the pairwise Spearman correlations between all of the arrays used in our experiment after quantile normalization and filtering out probes not meeting certain quality thresholds (see Methods). The clustering was based on a correlation-derived distance matrix. The color bar represents the three dates on which the samples were transfected. Samples clearly cluster based on the date on which they were transfected.
(TIF)

Figure S2 Diagnostic plots for RUV-2 normalization procedure. (a) The number of genes classified as differentially expressed (5% FDR; y-axis) in pairwise comparisons between the samples from the three transfection dates at different values of ‘k’ (x-axis). ‘k’ represents the number of factors regressed out of the expression estimates. No genes are detected as differentially expressed after regressing out two or more factors (k≥2). (b) The number of genes classified as differentially expressed in pairwise comparisons between the control samples (y-axis) from the three different transfection dates at a range of values for ‘k’ (x-axis). We repeated knockdowns for two factors on different transfection dates and so we used these replicate experiments to evaluate the effects of RUV-2. (c) The number of genes classified as differentially expressed in common between the two experiments where we knocked down *IRF5* (y-axis) at different values of ‘k’ (x-axis). (d) The number of genes classified as differentially expressed in common between the two experiments where we knocked down *SP1* (y-axis) at different values of ‘k’ (x-axis). (e) Correlation of −Log_10_(P-values) for the two *IRF5* experiments (y-axis) at different values of ‘k’ (x-axis). (f) Correlation of −Log_10_(P-values) for the two *SP1* experiments (y-axis) at different values of ‘k’ (x-axis). The dashed red lines in (c–f) highlight the results for k = 8, the value we ultimately chose for our normalization.
(TIF)

Figure S3 Heatmap of microarray correlations after RUV-2 correction. A heatmap showing the pairwise Spearman correlations between all of the arrays used in our experiment after quantile normalization, RUV-2 correction and filtering out probes not meeting certain quality thresholds (see Methods). The clustering was based on a correlation-derived distance matrix. The color bar represents the three dates on which the samples were transfected. Samples no longer cluster based on the date on which they were transfected after RUV-2 correction.
(TIF)

Figure S4 RLE plots for microarrays in our experiment. RLE plots can be used to identify a bias or increased variance in probe intensities for each microarray in an experiment. For each probe on the array, the difference between the probe intensity for a particular microarray and the median intensity across all microarrays is calculated. The deviations for all probes on the array are then visualized with a boxplot. A systematic shift from 0 would indicate a bias in expression estimates the array, while an increased interquartile range would indicate increased variance in probe intensities compared to the global median. Arrays in (a) have been quantile normalized. The colors of the boxes indicate the date of transcription for that particular array. There are no obvious biases for any arrays and overall the variance is low for all arrays. However, there is clearly increased variance for the “batch 3” arrays compared to the others. Arrays in (b) have been quantile normalized and RUV-2 corrected. All samples are now more centered on 0 and have smaller interquartile ranges. In addition, the variance is more consistent across all arrays than when the arrays were only quantile normalized.
(TIF)

Figure S5 Principal components analysis of the arrays before and after RUV-2 correction. (a) The first and second principal components (x- and y-axis, respectively) appear correlated with the three transfection dates even after quantile normalizing the data (as indicated by the color scheme). (b) The first two principal components do not appear correlated with transfection date following RUV-2 correction. The three samples in the upper right hand corner are the three replicates for the knockdown of *IRF4*.
(TIF)

Figure S6 Number of genes expressed in each knockdown experiment. Barplot of the number of genes detected as expressed in each knockdown experiment. To be classified as expressed, the probe for the gene had to have a detection p-value less than 0.01 in either all of the knockdown triplicates or all 18 control samples. The color scheme identifies which date the transfection took place on.
(TIF)

Figure S7 Distributions of factor binding from the perspective of (a) the factor and (b) the target gene. Histograms showing the distribution of (a) the number of genes expressed in at least one experiment that were bound (binding +/−10 kb form the TSS) by each factor that was differentially expressed in at least one knockdown experiment (N = 131) and (b) the number of factors binding each gene (N = 8,872).
(TIF)

Figure S8 Effect of paralogs on likelihood of differential expression in knockdown experiments. (a) For the 29 factors for which we had both direct binding data and expression data, boxplot of fraction of bound targets differentially expressed in the knockdown experiment stratified by number of paralogs each transcription factor has. (b) Fraction of direct targets differentially expressed in the knockdown plotted against the percent identity of the nearest paralog for that factor.
(TIF)

Table S1 Summary of factors targeted by siRNAs in our knockdown experiments. This table reports summary information for the 112 factors we targeted by siRNA. The columns are as follows: Column 1: “Gene name” – Name of factor targeted by the siRNA. Column 2: “ensGene” – Ensembl gene ID for the factor targeted. Column 3–4: “qPCR Forward Primer” & “qPCR Reverse Primer” – Sequence of primers used to evaluate the knockdown level by qPCR. Column 5: “Screen Kd %” – Knockdown level achieved at 48 hrs post-transfection for the targeted factor in the screening phase of our experiment. The knockdown level is relative to a control sample transfected in parallel with a pool of non-targeting siRNAs. Column 6: “Final Transfection Kd% (Ave. of Replicates)” – Knockdown level of the samples transfected for our experiment as measured by qPCR on RNA taken from the samples 48 hrs post-transfection. The knockdown level is relative to a control sample transfected in parallel with a pool of non-targeting siRNAs. Column 7: “Experimental Transfection Date” – The date on which the samples were transfected with siRNA. Samples were transfected on one of three different dates. Column 8: “Microarray Hybridization Date” – The date on which the samples were hybridized on Illumina HT-12 arrays. Samples were hybridized on one of three different dates.
(XLSX)

Table S2 Significance of correlations between principal components and several confounding variables. This table reports the significance of associations between different principal components and several potential confounding variables. The row labeled ‘PCImportance’ reports the fraction of variance captured by each component.
(TXT)

Table S3 Summary of results. This table reports the summary of our differential expression and binding data analysis for all of the genes expressed in the experiments. Below is a key for the columns included in the file: Column 1: “ProbeID” – Illumina probe identifier for each gene included in our analysis. Column 2: “ENSGID” – Ensembl gene ID for each gene. Column 3: “Symbol” – Gene symbol for each gene. Columns 3–62: “[GeneSymbol]_[ENSGID]_DE_p” – Each of these columns report the P-values from our likelihood-ratio test comparing the knockdown samples to the controls for one of the knockdown experiments. Columns 63–263: “[GeneSymbol]_[ENSGID]_binding” – Each of these columns reports the binding data for the 201 factors for which we had some binding data. For each row, the counts represent the number of binding events within 10 kb of the transcription start site observed for that factor. Only 138 of the 201 factors had a usable probe and were detected as expressed on our arrays, and only 131 were differentially expressed in at least one knockdown experiment.
(ZIP)

Table S4 Gene Ontology enrichments for DE genes in the knockdown experiments. This table reports the GO categories that are enriched among differentially expressed genes for each knockdown (P-values were adjusted for multiple testing with the “BH” method of the ‘p.adjust’ function in R). Each row is a GO category that is significant in at least one knockdown. Each column represents a knockdown. A value of ‘NA’ indicates that the GO category was not significant in the knockdown. This could be either because the category was not enriched or because it did not have enough annotated genes in a particular knockdown.
(TXT)

Table S5 BED file reporting the binding events used in our analysis. This zipped BED file contains the midpoint for all binding event we used in our analysis. To obtain binding information, we combined DNase-seq binding data for all motifs linked to a given factor by TRANSFAC and any ENCODE ChIP-seq data for the factor, took the union of all binding events and then calculated the midpoint for each BED record using pybedtools. The file includes 2,038,283 binding events for 201 different factors. The columns included in this file are: Column 1 – Chromosome. Column 2 – Midpoint of binding event. Column 3 – Midpoint of binding event repeated. Column 4 – Gene symbol of bound factor.
(ZIP)

Table S6 Genes classified as functionally bound based on different window sizes about the TSS. For various window sizes about the transcription start site (from 1 kb to 20 kb) the fraction of bound genes that were also classified as differentially expressed was calculated.
(PDF)

Table S7 Summary of overlap of binding and differential expression using ENCODE's DNase-based assignments of target genes for regulatory regions. Using DNase-based assignments of target genes for intergenic regions genome-wide, we assessed the overlap between factor binding and differential expression of factor binding in the knockdowns. The results are summarized in this table.
(TXT)

## References

[1] Jolma A, Yan J, Whitington T, Toivonen J, Nitta KR, et al (2013) DNA-binding specificities of human transcription factors. Cell 152: 327–339 doi:10.1016/j.cell.2012.12.009 23332764 10.1016/j.cell.2012.12.009

[2] Nobrega MA, Ovcharenko I, Afzal V, Rubin EM (2003) Scanning human gene deserts for long-range enhancers. Science 302: 413 doi:10.1126/science.1088328 14563999 10.1126/science.1088328

[3] Bernstein BE, Birney E, Dunham I, Green ED, Gunter C, et al (2012) An integrated encyclopedia of DNA elements in the human genome. Nature 489: 57–74 doi:10.1038/nature11247 22955616 10.1038/nature11247 PMC3439153

[4] Pique-Regi R, Degner JF, Pai AA, Gaffney DJ, Gilad Y, et al (2011) Accurate inference of transcription factor binding from DNA sequence and chromatin accessibility data. Genome Res 21: 447–455 doi:10.1101/gr.112623.110 21106904 10.1101/gr.112623.110 PMC3044858

[5] Song L, Crawford GE (2010) DNase-seq: a high-resolution technique for mapping active gene regulatory elements across the genome from mammalian cells. Cold Spring Harb Protoc 2010: pdb.prot5384 doi:10.1101/pdb.prot5384 20150147 10.1101/pdb.prot5384 PMC3627383

[6] Yan J, Enge M, Whitington T, Dave K, Liu J, et al (2013) Transcription factor binding in human cells occurs in dense clusters formed around cohesin anchor sites. Cell 154: 801–813 doi:10.1016/j.cell.2013.07.034 23953112 10.1016/j.cell.2013.07.034

[7] Gaffney DJ, Veyrieras J-B, Degner JF, Pique-Regi R, Pai AA, et al (2012) Dissecting the regulatory architecture of gene expression QTLs. Genome Biol 13: R7 doi:10.1186/gb-2012-13-1-r7 22293038 10.1186/gb-2012-13-1-r7 PMC3334587

[8] Bell JT, Pai AA, Pickrell JK, Gaffney DJ, Pique-Regi R, et al (2011) DNA methylation patterns associate with genetic and gene expression variation in HapMap cell lines. Genome Biol 12: R10 doi:10.1186/gb-2011-12-1-r10 21251332 10.1186/gb-2011-12-1-r10 PMC3091299

[9] Degner JF, Pai AA, Pique-Regi R, Veyrieras J-B, Gaffney DJ, et al (2012) DNase I sensitivity QTLs are a major determinant of human expression variation. Nature 482: 390–394 doi:10.1038/nature10808 22307276 10.1038/nature10808 PMC3501342

[10] Spivakov M, Akhtar J, Kheradpour P, Beal K, Girardot C, et al (2012) Analysis of variation at transcription factor binding sites in Drosophila and humans. Genome Biol 13: R49 doi:10.1186/gb-2012-13-9-r49 22950968 10.1186/gb-2012-13-9-r49 PMC3491393

[11] Ernst J, Kheradpour P, Mikkelsen TS, Shoresh N, Ward LD, et al (2011) Mapping and analysis of chromatin state dynamics in nine human cell types. Nature 473: 43–49 doi:10.1038/nature09906 21441907 10.1038/nature09906 PMC3088773

[12] Gilad Y, Rifkin SA, Pritchard JK (2008) Revealing the architecture of gene regulation: the promise of eQTL studies. Trends Genet 24: 408–415 doi:10.1016/j.tig.2008.06.001 18597885 10.1016/j.tig.2008.06.001 PMC2583071

[13] Chia N-Y, Chan Y-S, Feng B, Lu X, Orlov YL, et al (2010) A genome-wide RNAi screen reveals determinants of human embryonic stem cell identity. Nature 468: 316–320 doi:10.1038/nature09531 20953172 10.1038/nature09531

[14] Yang A, Zhu Z, Kapranov P, McKeon F, Church GM, et al (2006) Relationships between p63 binding, DNA sequence, transcription activity, and biological function in human cells. Mol Cell 24: 593–602 doi:10.1016/j.molcel.2006.10.018 17188034 10.1016/j.molcel.2006.10.018

[15] Krig SR, Jin VX, Bieda MC, O'Geen H, Yaswen P, et al (2007) Identification of genes directly regulated by the oncogene ZNF217 using chromatin immunoprecipitation (ChIP)-chip assays. J Biol Chem 282: 9703–9712 doi:10.1074/jbc.M611752200 17259635 10.1074/jbc.M611752200 PMC2269729

[16] Xu X, Bieda M, Jin VX, Rabinovich A, Oberley MJ, et al (2007) A comprehensive ChIP-chip analysis of E2F1, E2F4, and E2F6 in normal and tumor cells reveals interchangeable roles of E2F family members. Genome Res 17: 1550–1561 doi:10.1101/gr.6783507 17908821 10.1101/gr.6783507 PMC2045138

[17] Kawaji H, Severin J, Lizio M, Waterhouse A, Katayama S, et al (2009) The FANTOM web resource: from mammalian transcriptional landscape to its dynamic regulation. Genome Biol 10: R40 doi:10.1186/gb-2009-10-4-r40 19374775 10.1186/gb-2009-10-4-r40 PMC2688931

[18] Suzuki H, Forrest ARR, van Nimwegen E, Daub CO, Balwierz PJ, et al (2009) The transcriptional network that controls growth arrest and differentiation in a human myeloid leukemia cell line. Nat Genet 41: 553–562 doi:10.1038/ng.375 19377474 10.1038/ng.375 PMC6711855

[19] Cheng C, Alexander R, Min R, Leng J, Yip KY, et al (2012) Understanding transcriptional regulation by integrative analysis of transcription factor binding data. Genome Res 22: 1658–1667 doi:10.1101/gr.136838.111 22955978 10.1101/gr.136838.111 PMC3431483

[20] Gerstein MB, Kundaje A, Hariharan M, Landt SG, Yan K-K, et al (2012) Architecture of the human regulatory network derived from ENCODE data. Nature 489: 91–100 doi:10.1038/nature11245 22955619 10.1038/nature11245 PMC4154057

[21] Alemán LM, Doench J, Sharp PA (2007) Comparison of siRNA-induced off-target RNA and protein effects. RNA 13: 385–395 doi:10.1261/rna.352507 17237357 10.1261/rna.352507 PMC1800510

[22] De Candia P, Blekhman R, Chabot AE, Oshlack A, Gilad Y (2008) A combination of genomic approaches reveals the role of FOXO1a in regulating an oxidative stress response pathway. PLoS One 3: e1670 doi:10.1371/journal.pone.0001670 18301748 10.1371/journal.pone.0001670 PMC2244703

[23] Tamura T, Yanai H, Savitsky D, Taniguchi T (2008) The IRF family transcription factors in immunity and oncogenesis. Annu Rev Immunol 26: 535–584 doi:10.1146/annurev.immunol.26.021607.090400 18303999 10.1146/annurev.immunol.26.021607.090400

[24] Ashburner M, Ball CA, Blake JA, Botstein D, Butler H, et al (2000) Gene ontology: tool for the unification of biology. The Gene Ontology Consortium. Nat Genet 25: 25–29 doi:10.1038/75556 10802651 10.1038/75556 PMC3037419

[25] Kanno Y, Levi B-Z, Tamura T, Ozato K (2005) Immune cell-specific amplification of interferon signaling by the IRF-4/8-PU.1 complex. J Interferon Cytokine Res 25: 770–779 doi:10.1089/jir.2005.25.770 16375605 10.1089/jir.2005.25.770

[26] Tsuno T, Mejido J, Zhao T, Schmeisser H, Morrow A, et al (2009) IRF9 is a key factor for eliciting the antiproliferative activity of IFN-alpha. J Immunother 32: 803–816 doi:10.1097/CJI.0b013e3181ad4092 19752753 10.1097/CJI.0b013e3181ad4092 PMC2796296

[27] Eberlé D, Hegarty B, Bossard P, Ferré P, Foufelle F (2004) SREBP transcription factors: master regulators of lipid homeostasis. Biochimie 86: 839–848 doi:10.1016/j.biochi.2004.09.018 15589694 10.1016/j.biochi.2004.09.018

[28] Siepel A, Bejerano G, Pedersen JS, Hinrichs AS, Hou M, et al (2005) Evolutionarily conserved elements in vertebrate, insect, worm, and yeast genomes. Genome Res 15: 1034–1050 doi:10.1101/gr.3715005 16024819 10.1101/gr.3715005 PMC1182216

[29] Graur D, Zheng Y, Price N, Azevedo RBR, Zufall RA, et al (2013) On the immortality of television sets: “function” in the human genome according to the evolution-free gospel of ENCODE. Genome Biol Evol 5: 578–590 doi:10.1093/gbe/evt028 23431001 10.1093/gbe/evt028 PMC3622293

[30] Gitter A, Siegfried Z, Klutstein M, Fornes O, Oliva B, et al (2009) Backup in gene regulatory networks explains differences between binding and knockout results. Mol Syst Biol 5: 276 doi:10.1038/msb.2009.33 19536199 10.1038/msb.2009.33 PMC2710864

[31] Vilella AJ, Severin J, Ureta-Vidal A, Heng L, Durbin R, et al (2009) EnsemblCompara GeneTrees: Complete, duplication-aware phylogenetic trees in vertebrates. Genome Res 19: 327–335 doi:10.1101/gr.073585.107 19029536 10.1101/gr.073585.107 PMC2652215

[32] Latchman DS (2001) Transcription factors: bound to activate or repress. Trends Biochem Sci 26: 211–213 doi:10.1016/S0968-0004(01)01812-6 11295539 10.1016/s0968-0004(01)01812-6

[33] Boyle P, Després C (2010) Dual-function transcription factors and their entourage: unique and unifying themes governing two pathogenesis-related genes. Plant Signal Behav 5: 629–634. 20383056 10.4161/psb.5.6.11570 PMC3001550

[34] Hobert O, Jallal B, Ullrich A (1996) Interaction of Vav with ENX-1, a putative transcriptional regulator of homeobox gene expression. Mol Cell Biol 16: 3066–3073. 8649418 10.1128/mcb.16.6.3066 PMC231301

[35] Hirai SI, Ryseck RP, Mechta F, Bravo R, Yaniv M (1989) Characterization of junD: a new member of the jun proto-oncogene family. EMBO J 8: 1433–1439. 2504580 10.1002/j.1460-2075.1989.tb03525.x PMC400971

[36] Farnham PJ (2009) Insights from genomic profiling of transcription factors. Nat Rev Genet 10: 605–616 doi:10.1038/nrg2636 19668247 10.1038/nrg2636 PMC2846386

[37] Biggin MD (2011) Animal transcription networks as highly connected, quantitative continua. Dev Cell 21: 611–626 doi:10.1016/j.devcel.2011.09.008 22014521 10.1016/j.devcel.2011.09.008

[38] Thurman RE, Rynes E, Humbert R, Vierstra J, Maurano MT, et al (2012) The accessible chromatin landscape of the human genome. Nature 489: 75–82 doi:10.1038/nature11232 22955617 10.1038/nature11232 PMC3721348

[39] Wang Z, Gerstein M, Snyder M (2009) RNA-Seq: a revolutionary tool for transcriptomics. Nat Rev Genet 10: 57–63 doi:10.1038/nrg2484 19015660 10.1038/nrg2484 PMC2949280

[40] Peirson SN (2003) Experimental validation of novel and conventional approaches to quantitative real-time PCR data analysis. Nucleic Acids Res 31: 73e–73 doi:10.1093/nar/gng073 10.1093/nar/gng073 PMC167648 12853650

[41] Li H, Durbin R (2009) Fast and accurate short read alignment with Burrows-Wheeler transform. Bioinformatics 25: 1754–1760 doi:10.1093/bioinformatics/btp324 19451168 10.1093/bioinformatics/btp324 PMC2705234

[42] Abecasis GR, Altshuler D, Auton A, Brooks LD, Durbin RM, et al (2010) A map of human genome variation from population-scale sequencing. Nature 467: 1061–1073 doi:10.1038/nature09534 20981092 10.1038/nature09534 PMC3042601

[43] Du P, Kibbe WA, Lin SM (2008) lumi: a pipeline for processing Illumina microarray. Bioinformatics 24: 1547–1548 doi:10.1093/bioinformatics/btn224 18467348 10.1093/bioinformatics/btn224

[44] Gentleman RC, Carey VJ, Bates DM, Bolstad B, Dettling M, et al (2004) Bioconductor: open software development for computational biology and bioinformatics. Genome Biol 5: R80 doi:10.1186/gb-2004-5-10-r80 15461798 10.1186/gb-2004-5-10-r80 PMC545600

[45] Gagnon-Bartsch JA, Speed TP (2012) Using control genes to correct for unwanted variation in microarray data. Biostatistics 13: 539–552 doi:10.1093/biostatistics/kxr034 22101192 10.1093/biostatistics/kxr034 PMC3577104

[46] Smyth GK (2004) Linear models and empirical bayes methods for assessing differential expression in microarray experiments. Stat Appl Genet Mol Biol 3: Article3 doi:10.2202/1544-6115.1027 16646809 10.2202/1544-6115.1027

[47] Bolstad B, Irizarry R, Åstrand M, Speed T (2003) A comparison of normalization methods for high density oligonucleotide array data based on variance and bias. Bioinformatics 19: 185–193. 12538238 10.1093/bioinformatics/19.2.185

[48] Brettschneider J, Collin F, Bolstad BM, Speed TP (2008) Quality assessment for short oligonucleotide microarray data. Technometrics 50: 241–264 doi:10.1198/004017008000000334

[49] Storey JD, Tibshirani R (2003) Statistical significance for genomewide studies. Proc Natl Acad Sci 100: 9440–9445 doi:10.1073/pnas.1530509100 12883005 10.1073/pnas.1530509100 PMC170937

[50] Djebali S, Davis CA, Merkel A, Dobin A, Lassmann T, et al (2012) Landscape of transcription in human cells. Nature 489: 101–108 doi:10.1038/nature11233 22955620 10.1038/nature11233 PMC3684276

[51] Matys V, Kel-Margoulis O V, Fricke E, Liebich I, Land S, et al (2006) TRANSFAC and its module TRANSCompel: transcriptional gene regulation in eukaryotes. Nucleic Acids Res 34: D108–10 doi:10.1093/nar/gkj143 16381825 10.1093/nar/gkj143 PMC1347505

[52] Quinlan AR, Hall IM (2010) BEDTools: a flexible suite of utilities for comparing genomic features. Bioinformatics 26: 841–842 doi:10.1093/bioinformatics/btq033 20110278 10.1093/bioinformatics/btq033 PMC2832824

[53] Dale RK, Pedersen BS, Quinlan AR (2011) Pybedtools: a flexible Python library for manipulating genomic datasets and annotations. Bioinformatics 27: 3423–3424 doi:10.1093/bioinformatics/btr539 21949271 10.1093/bioinformatics/btr539 PMC3232365

[54] Neph S, Kuehn MS, Reynolds AP, Haugen E, Thurman RE, et al (2012) BEDOPS: high-performance genomic feature operations. Bioinformatics 28: 1919–1920 doi:10.1093/bioinformatics/bts277 22576172 10.1093/bioinformatics/bts277 PMC3389768

[55] Edgar R (2002) Gene Expression Omnibus: NCBI gene expression and hybridization array data repository. Nucleic Acids Res 30: 207–210 doi:10.1093/nar/30.1.207 11752295 10.1093/nar/30.1.207 PMC99122

