# Supplementary figures and images for: The Functional Consequences of Variation in Transcription Factor Binding
Source: PLoS Genet. 2014 Mar 6;10(3):e1004226. doi: 10.1371/journal.pgen.1004226 (PMC3945204; doi:10.1371/journal.pgen.1004226)

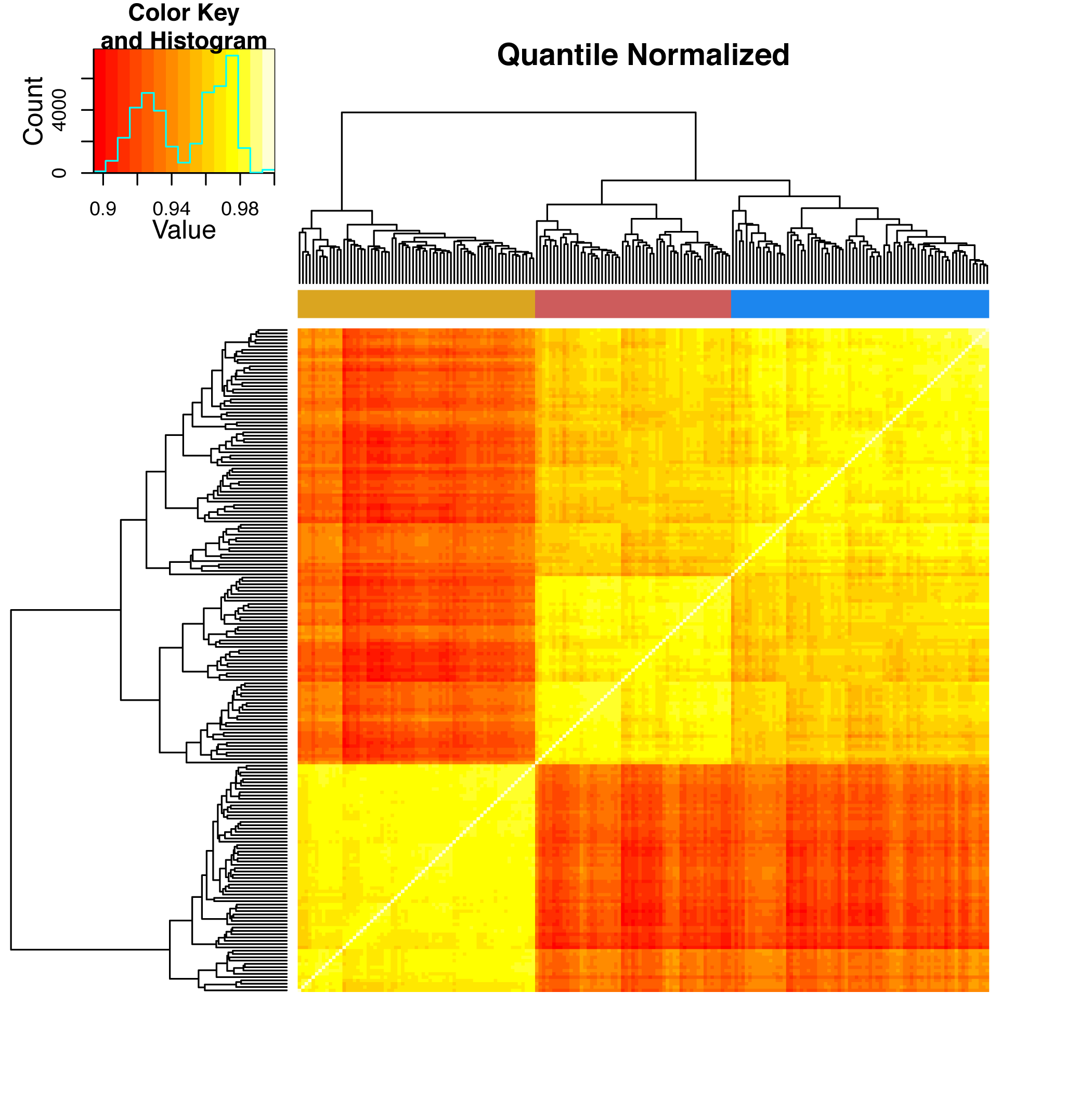

Supplement: Figure S1 — Heatmap of microarray correlations. A heatmap showing the pairwise Spearman correlations between all of the arrays used in our experiment after quantile normalization and filtering out probes not meeting certain quality thresholds (see Methods). The clustering was based on a correlation-derived distance matrix. The color bar represents the three dates on which the samples were transfected. Samples clearly cluster based on the date on which they were transfected. (TIF) [file pgen.1004226.s001.tif]

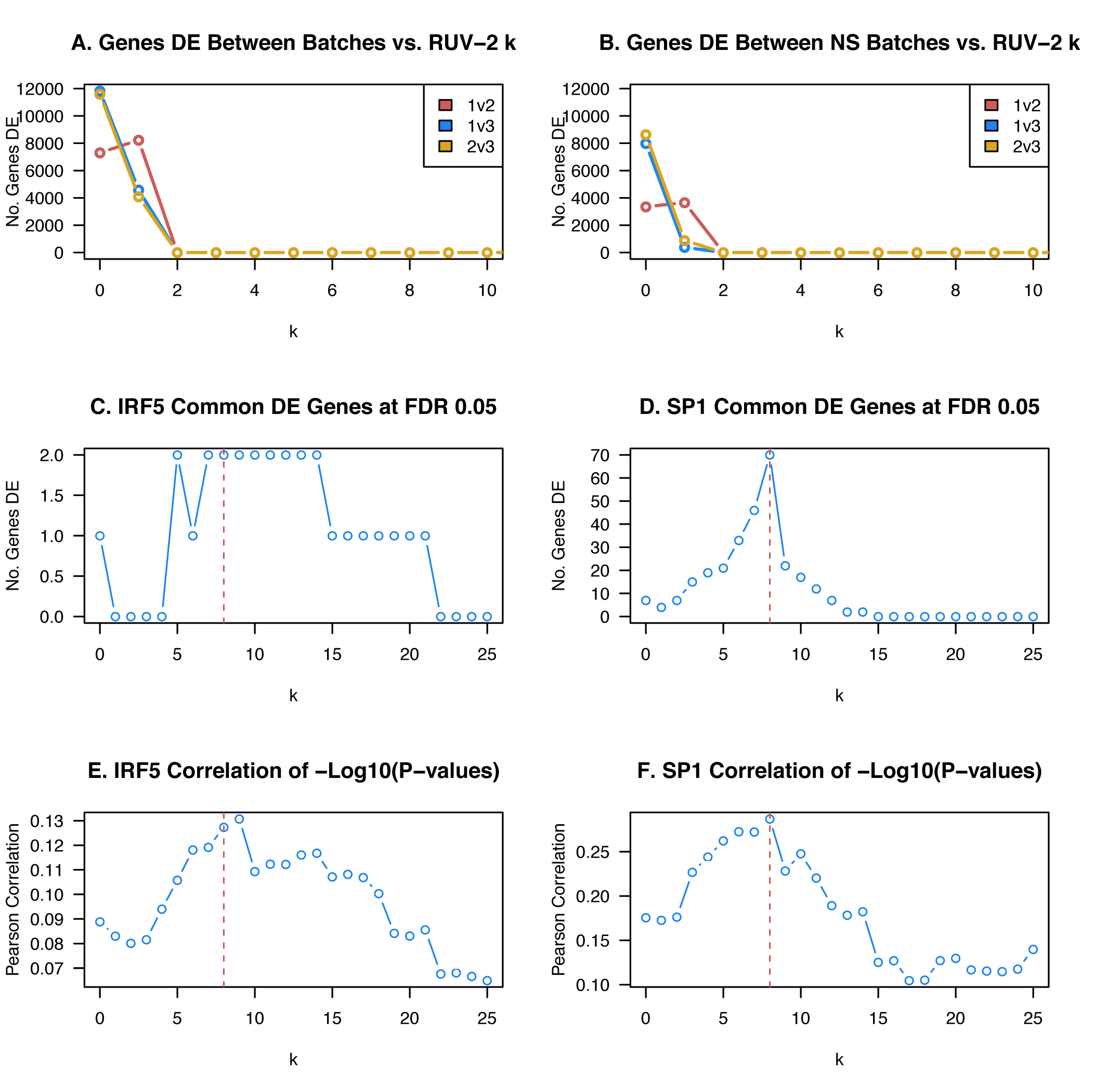

Supplement: Figure S2 — Diagnostic plots for RUV-2 normalization procedure. (a) The number of genes classified as differentially expressed (5% FDR; y-axis) in pairwise comparisons between the samples from the three transfection dates at different values of ‘k’ (x-axis). ‘k’ represents the number of factors regressed out of the expression estimates. No genes are detected as differentially expressed after regressing out two or more factors (k≥2). (b) The number of genes classified as differentially expressed in pairwise comparisons between the control samples (y-axis) from the three different transfection dates at a range of values for ‘k’ (x-axis). We repeated knockdowns for two factors on different transfection dates and so we used these replicate experiments to evaluate the effects of RUV-2. (c) The number of genes classified as differentially expressed in common between the two experiments where we knocked down IRF5 (y-axis) at different values of ‘k’ (x-axis). (d) The number of genes classified as differentially expressed in common between the two experiments where we knocked down SP1 (y-axis) at different values of ‘k’ (x-axis). (e) Correlation of −Log10(P-values) for the two IRF5 experiments (y-axis) at different values of ‘k’ (x-axis). (f) Correlation of −Log10(P-values) for the two SP1 experiments (y-axis) at different values of ‘k’ (x-axis). The dashed red lines in (c–f) highlight the results for k = 8, the value we ultimately chose for our normalization. (TIF) [file pgen.1004226.s002.tif]

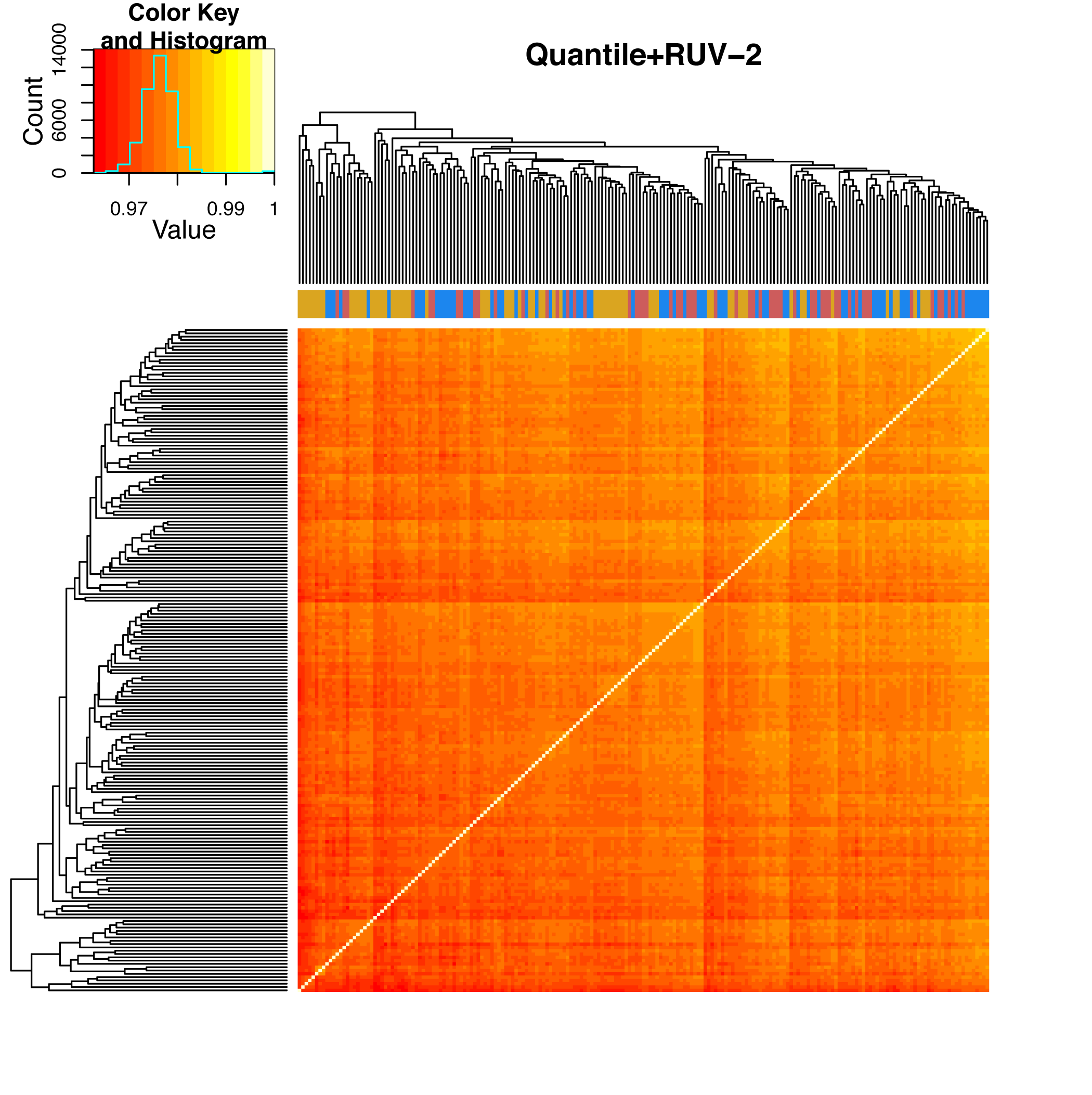

Supplement: Figure S3 — Heatmap of microarray correlations after RUV-2 correction. A heatmap showing the pairwise Spearman correlations between all of the arrays used in our experiment after quantile normalization, RUV-2 correction and filtering out probes not meeting certain quality thresholds (see Methods). The clustering was based on a correlation-derived distance matrix. The color bar represents the three dates on which the samples were transfected. Samples no longer cluster based on the date on which they were transfected after RUV-2 correction. (TIF) [file pgen.1004226.s003.tif]

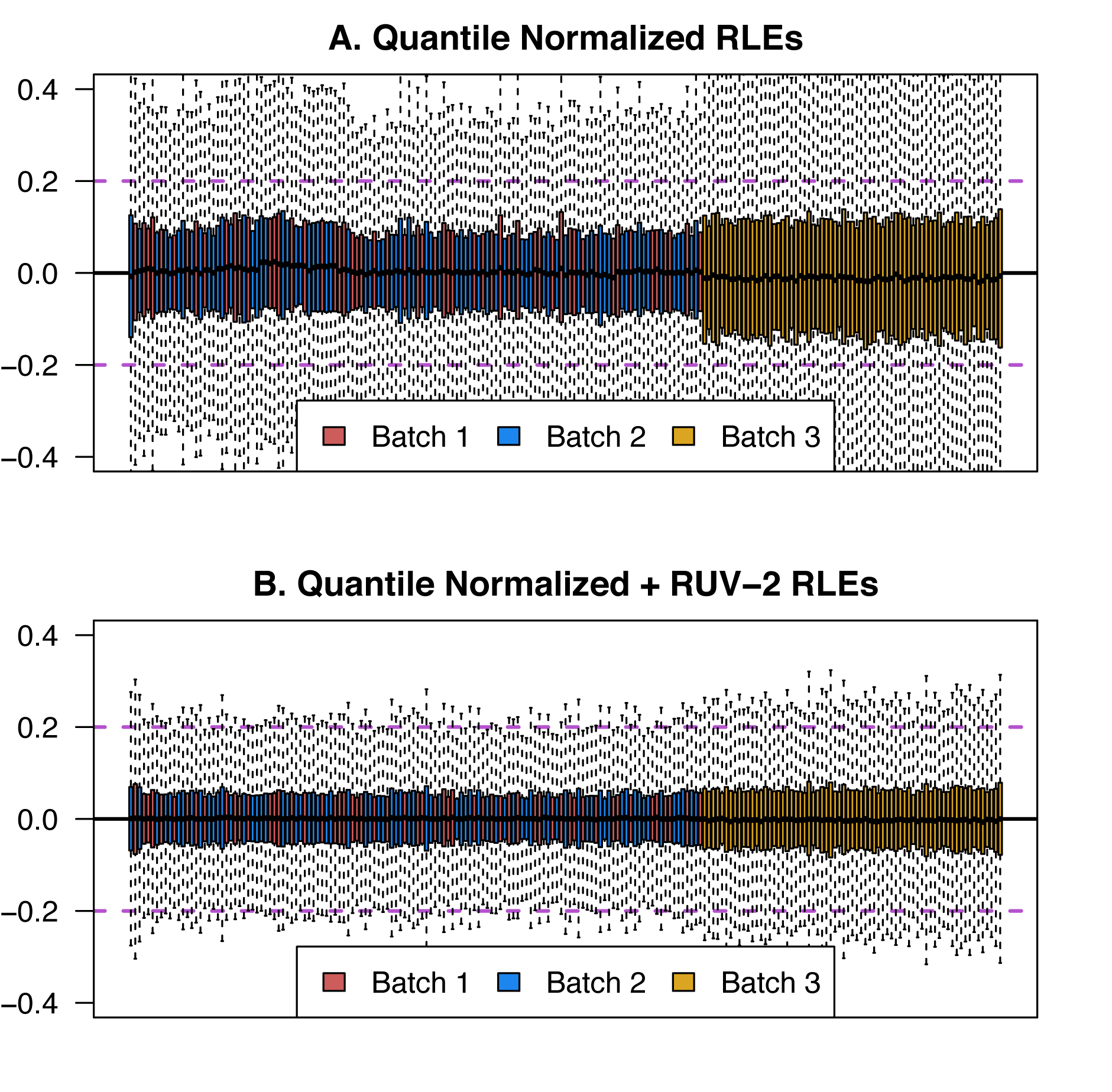

Supplement: Figure S4 — RLE plots for microarrays in our experiment. RLE plots can be used to identify a bias or increased variance in probe intensities for each microarray in an experiment. For each probe on the array, the difference between the probe intensity for a particular microarray and the median intensity across all microarrays is calculated. The deviations for all probes on the array are then visualized with a boxplot. A systematic shift from 0 would indicate a bias in expression estimates the array, while an increased interquartile range would indicate increased variance in probe intensities compared to the global median. Arrays in (a) have been quantile normalized. The colors of the boxes indicate the date of transcription for that particular array. There are no obvious biases for any arrays and overall the variance is low for all arrays. However, there is clearly increased variance for the “batch 3” arrays compared to the others. Arrays in (b) have been quantile normalized and RUV-2 corrected. All samples are now more centered on 0 and have smaller interquartile ranges. In addition, the variance is more consistent across all arrays than when the arrays were only quantile normalized. (TIF) [file pgen.1004226.s004.tif]

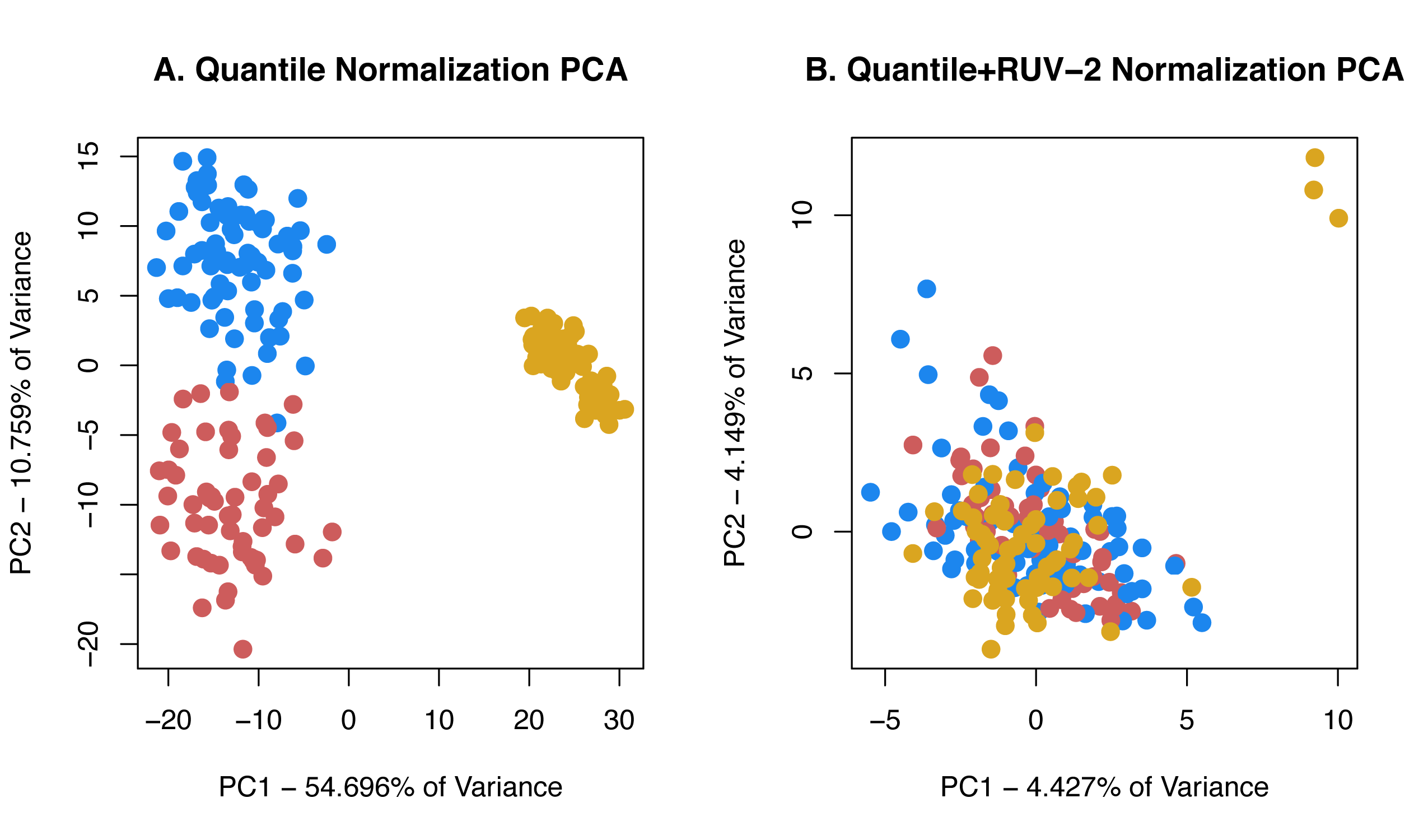

Supplement: Figure S5 — Principal components analysis of the arrays before and after RUV-2 correction. (a) The first and second principal components (x- and y-axis, respectively) appear correlated with the three transfection dates even after quantile normalizing the data (as indicated by the color scheme). (b) The first two principal components do not appear correlated with transfection date following RUV-2 correction. The three samples in the upper right hand corner are the three replicates for the knockdown of IRF4. (TIF) [file pgen.1004226.s005.tif]

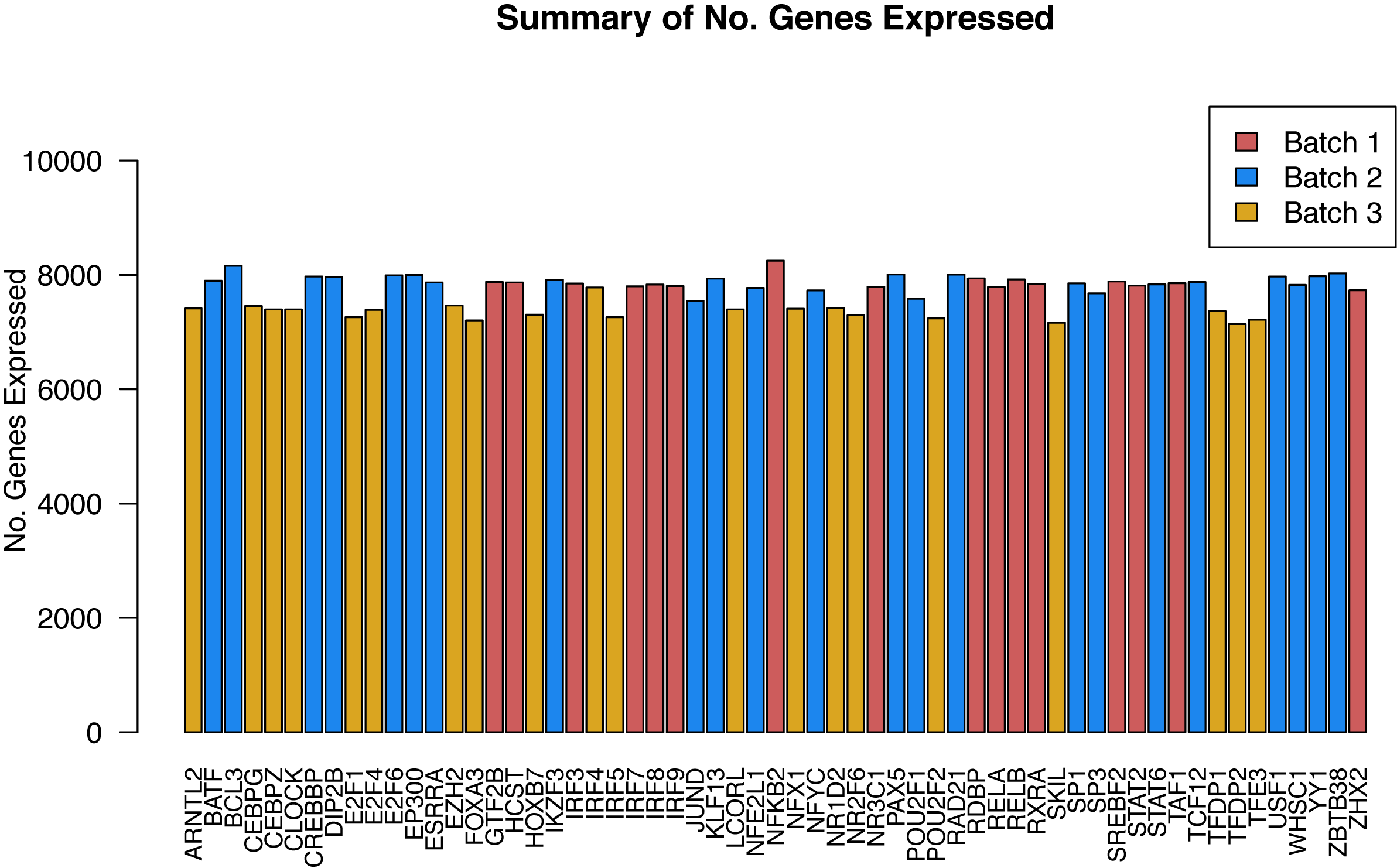

Supplement: Figure S6 — Number of genes expressed in each knockdown experiment. Barplot of the number of genes detected as expressed in each knockdown experiment. To be classified as expressed, the probe for the gene had to have a detection p-value less than 0.01 in either all of the knockdown triplicates or all 18 control samples. The color scheme identifies which date the transfection took place on. (TIF) [file pgen.1004226.s006.tif]

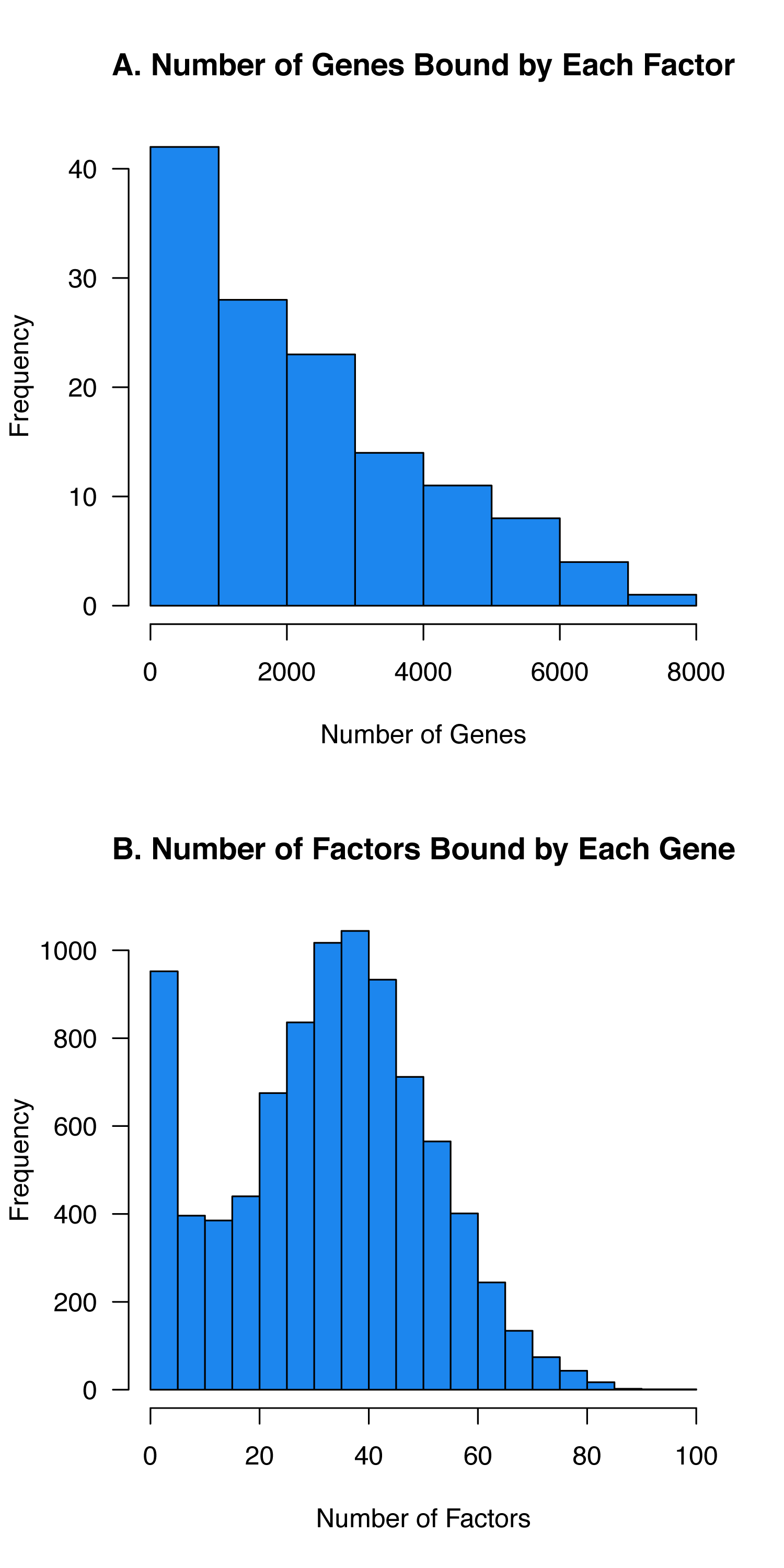

Supplement: Figure S7 — Distributions of factor binding from the perspective of (a) the factor and (b) the target gene. Histograms showing the distribution of (a) the number of genes expressed in at least one experiment that were bound (binding +/−10 kb form the TSS) by each factor that was differentially expressed in at least one knockdown experiment (N = 131) and (b) the number of factors binding each gene (N = 8,872). (TIF) [file pgen.1004226.s007.tif]

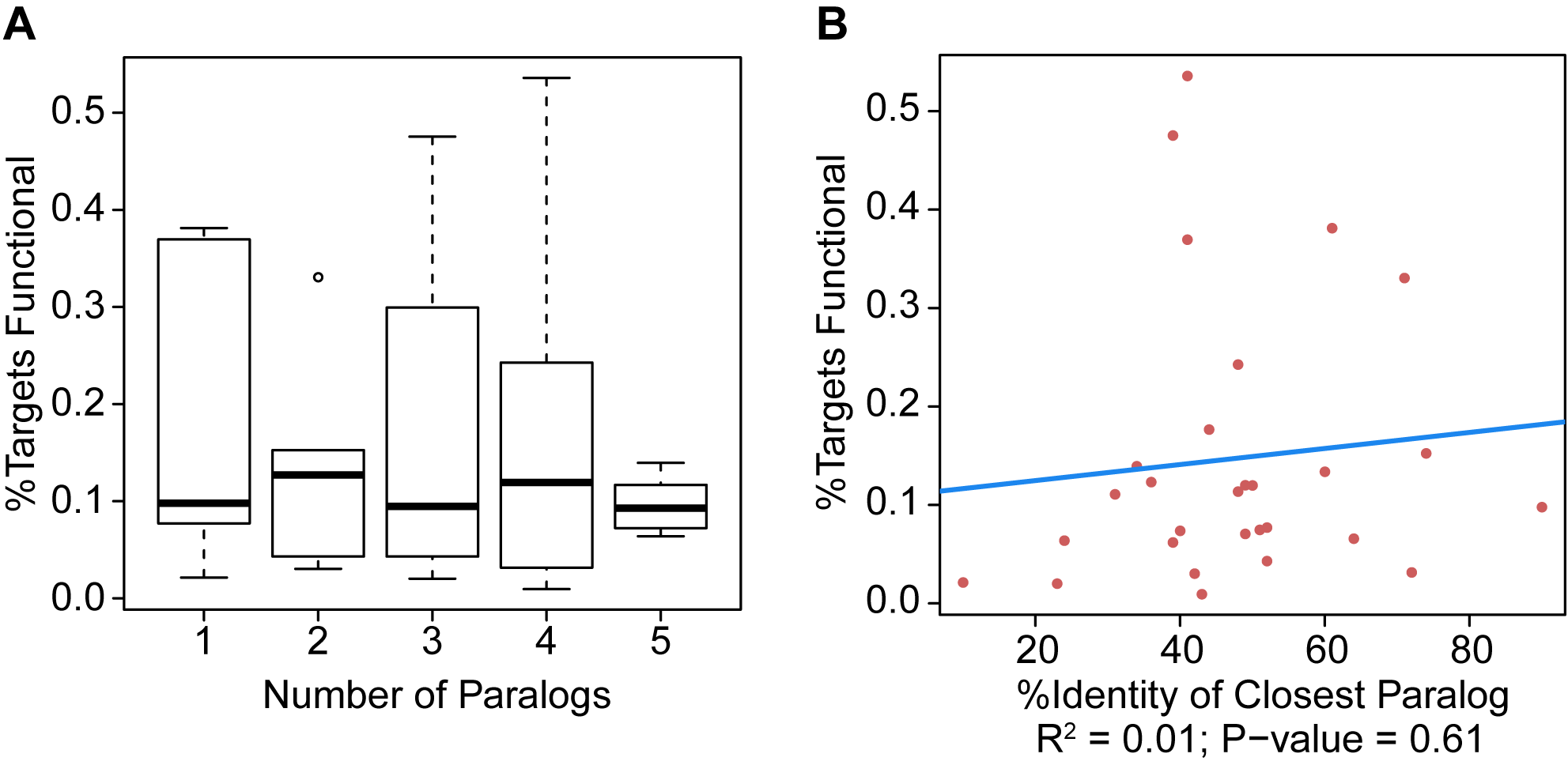

Supplement: Figure S8 — Effect of paralogs on likelihood of differential expression in knockdown experiments. (a) For the 29 factors for which we had both direct binding data and expression data, boxplot of fraction of bound targets differentially expressed in the knockdown experiment stratified by number of paralogs each transcription factor has. (b) Fraction of direct targets differentially expressed in the knockdown plotted against the percent identity of the nearest paralog for that factor. (TIF) [file pgen.1004226.s008.tif]
