# Supplementary material for: The Functional Consequences of Variation in Transcription Factor Binding
Source: PLoS Genet. 2014 Mar 6;10(3):e1004226. doi: 10.1371/journal.pgen.1004226 (PMC3945204; doi:10.1371/journal.pgen.1004226)
Supplement: Table S6 — Genes classified as functionally bound based on different window sizes about the TSS. For various window sizes about the transcription start site (from 1 kb to 20 kb) the fraction of bound genes that were also classified as differentially expressed was calculated. (PDF) [file pgen.1004226.s014.pdf]

			+/- 1kb from TSS			+/- 5kb from TSS			+/- 10kb from TSS			+/- 20kb from TSS		
Factor	Expressed Genes	DE Genes	Bound Genes	Functionally Bound	Percent Not Functional	Bound Genes	Functionally Bound	Percent Not Functional	Bound Genes	Functionally Bound	Percent Not Functional	Bound Genes	Functionally Bound	Percent Not Functional
ARNTL2	7414	772	330	33	90.0%	547	60	89.0%	695	79	88.6%	971	105	89.2%
BATF	7897	211	676	20	97.0%	1919	59	96.9%	2738	83	97.0%	3933	118	97.0%
BCL3	8158	532	929	55	94.1%	1672	112	93.3%	2271	145	93.6%	3178	199	93.7%
CLOCK	7394	522	333	24	92.8%	548	40	92.7%	693	49	92.9%	972	73	92.5%
E2F1	7259	452	565	36	93.6%	782	53	93.2%	962	71	92.6%	1286	86	93.3%
E2F4	7387	536	1588	107	93.3%	2088	148	92.9%	2474	185	92.5%	3003	225	92.5%
EP300	7999	2919	1210	439	63.7%	2031	771	62.0%	2628	1002	61.9%	3457	1311	62.1%
EZH2	7463	467	69	3	95.7%	161	12	92.5%	243	16	93.4%	381	23	94.0%
IRF3	7848	113	152	2	98.7%	203	3	98.5%	248	5	98.0%	315	6	98.1%
IRF4	7778	3892	1016	522	48.6%	1903	1015	46.7%	2564	1374	46.4%	3520	1865	47.0%
IRF8	7831	67	305	2	99.3%	796	9	98.9%	1274	12	99.1%	1962	14	99.3%
JUND	7547	324	114	7	93.9%	364	16	95.6%	627	27	95.7%	1022	45	95.6%
NFE2L1	7772	867	94	17	81.9%	256	36	85.9%	416	58	86.1%	736	84	88.6%
NFKB2	8249	1177	416	69	83.4%	792	144	81.8%	1098	194	82.3%	1597	273	82.9%
NFYC	7728	188	1071	25	97.7%	1645	35	97.9%	2110	45	97.9%	2760	67	97.6%
PAX5	8006	2483	2272	748	67.1%	3387	1147	66.1%	4156	1374	66.9%	5078	1663	67.3%
POU2F1	7581	688	343	39	88.6%	1111	129	88.4%	1713	190	88.9%	2635	287	89.1%
POU2F2	7238	859	2822	334	88.2%	3963	475	88.0%	4696	563	88.0%	5532	653	88.2%
RAD21	8005	2780	1204	449	62.7%	2622	1003	61.7%	3853	1424	63.0%	5457	1974	63.8%
RELA	7789	438	1251	87	93.0%	2127	138	93.5%	2742	170	93.8%	3605	229	93.6%
RXRA	7843	232	1037	30	97.1%	1833	57	96.9%	2377	75	96.8%	3227	106	96.7%
SP1	7850	3674	4041	1898	53.0%	5181	2456	52.6%	5812	2763	52.5%	6410	3018	52.9%
SP3	7677	877	2943	358	87.8%	3851	486	87.4%	4452	549	87.7%	5101	624	87.8%
TAF1	7854	800	3781	374	90.1%	4743	464	90.2%	5313	520	90.2%	5904	580	90.2%
TCF12	7874	958	3123	424	86.4%	4472	603	86.5%	5261	704	86.6%	6028	784	87.0%
TFDP1	7364	1178	186	38	79.6%	274	47	82.8%	341	52	84.8%	446	67	85.0%
TFDP2	7139	787	497	60	87.9%	689	86	87.5%	841	101	88.0%	1136	126	88.9%
USF1	7972	547	1702	142	91.7%	2483	196	92.1%	3047	235	92.3%	3773	279	92.6%
YY1	7976	1868	3858	947	75.5%	5136	1255	75.6%	5842	1418	75.7%	6573	1567	76.2%
Minimum					48.6%			46.7%			46.4%			47.0%
Maximum					99.3%			98.9%			99.1%			99.3%
Median					90.0%			89.0%			88.9%			89.2%
